# Recommendations for detection, validation, and evaluation of RNA editing events in cardiovascular and neurological/neurodegenerative diseases

**DOI:** 10.1016/j.omtn.2023.102085

**Published:** 2023-12-05

**Authors:** Korina Karagianni, Alessia Bibi, Alisia Madé, Shubhra Acharya, Mikko Parkkonen, Teodora Barbalata, Prashant K. Srivastava, David de Gonzalo-Calvo, Constanza Emanueli, Fabio Martelli, Yvan Devaux, Dimitra Dafou, A. Yaël Nossent, on behalf of EU-CardioRNA COST Action CA17129

**Affiliations:** 1 Department of Genetics, Development, and Molecular Biology, School of Biology, Aristotle University of Thessaloniki, 541 24 Thessaloniki, Greece; 2 Molecular Cardiology Laboratory, IRCCS Policlinico San Donato, Via Morandi 30, San Donato Milanese, 20097 Milan, Italy; 3 Department of Biosciences, University of Milan, Milan, Italy; 4 Cardiovascular Research Unit, Luxembourg Institute of Health, Strassen, Luxembourg; 5 Faculty of Science, Technology and Medicine, University of Luxembourg, Esch-sur-alzette, Luxembourg; 6 Research Unit of Biomedicine and Internal Medicine, Department of Pharmacology and Toxicology, University of Oulu, Oulu, Finland; 7 Lipidomics Department, Institute of Cellular Biology and Pathology “Nicolae Simionescu” of the Romanian Academy, 8, B. P. Hasdeu Street, 050568 Bucharest, Romania; 8 National Heart & Lung Institute, Imperial College London, London, UK; 9 Translational Research in Respiratory Medicine, University Hospital Arnau de Vilanova and Santa Maria, IRBLleida, Lleida, Spain; 10 CIBER of Respiratory Diseases (CIBERES), Institute of Health Carlos III, Madrid, Spain; 11 Department of Surgery, Leiden University Medical Center, Leiden, the Netherlands; 12 Department of Nutrition, Exercise and Sports (NEXS), University of Copenhagen, Copenhagen, Denmark

**Keywords:** MT: RNA/DNA editing, RNA editing, A-to-I editing, C-to-U editing, methodology, cardiovascular disease, neurovascular disease, neurodegenerative disease

## Abstract

RNA editing, a common and potentially highly functional form of RNA modification, encompasses two different RNA modifications, namely adenosine to inosine (A-to-I) and cytidine to uridine (C-to-U) editing. As inosines are interpreted as guanosines by the cellular machinery, both A-to-I and C-to-U editing change the nucleotide sequence of the RNA. Editing events in coding sequences have the potential to change the amino acid sequence of proteins, whereas editing events in noncoding RNAs can, for example, affect microRNA target binding. With advancing RNA sequencing technology, more RNA editing events are being discovered, studied, and reported. However, RNA editing events are still often overlooked or discarded as sequence read quality defects. With this position paper, we aim to provide guidelines and recommendations for the detection, validation, and follow-up experiments to study RNA editing, taking examples from the fields of cardiovascular and brain disease. We discuss all steps, from sample collection, storage, and preparation, to different strategies for RNA sequencing and editing-sensitive data analysis strategies, to validation and follow-up experiments, as well as potential pitfalls and gaps in the available technologies. This paper may be used as an experimental guideline for RNA editing studies in any disease context.

## Graphical abstract

**Figure:**
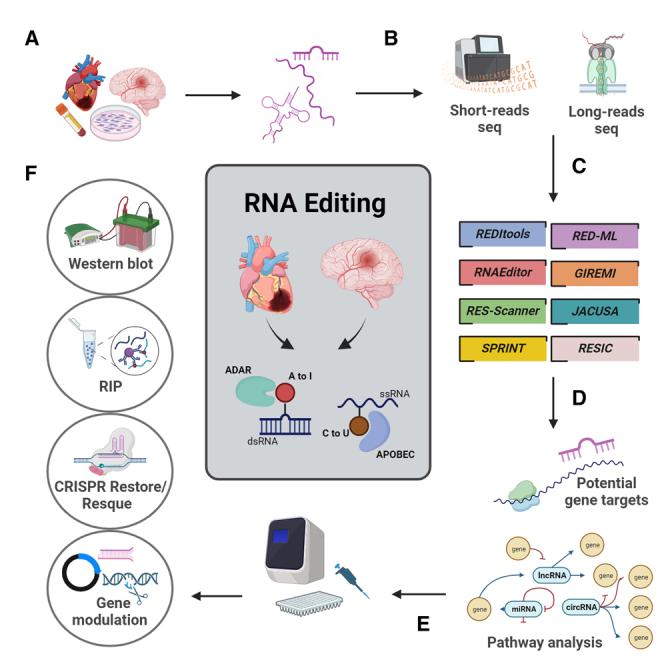

## Teaser

With this position paper, Nossent and colleagues provide guidelines for the detection, validation and follow-up experiments to study RNA editing. We discuss all steps, from sample collection, storage, and preparation, to different strategies for RNA sequencing and data analysis, to validation and follow-up experiments, as well as pitfalls in the available technologies.

## Introduction

RNA editing is a post-transcriptional modification that alters the sequence of an RNA transcript. It was first discovered by Benne et al. in a mitochondrion-encoded mRNA in *Trypanosoma brucei*.^1^ Wagner et al. showed that RNA editing also occurs in mammalian cells.^2^

In fact, two types of RNA editing were found in mammals, involving the enzymatic deamination of either adenosine to inosine (A-to-I) or cytidine to uridine (C-to-U) nucleotides in RNA.^2^^,^^3^^,^^4^ A-to-I, which is the most common form of RNA editing, is mediated by the ADAR (adenosine deaminases acting on RNA) family of enzymes, ADAR1, ADAR2, and ADAR3.^5^^,^^6^ ADAR1 and ADAR2 are catalytically active. ADAR1 is ubiquitously expressed while ADAR2 expression is very low in some tissues. ADAR3 has no proven catalytic activity, and its expression is limited to the brain.^7^ ADAR3 has been shown to inhibit ADAR1-induced RNA editing.^8^ ADAR1 and ADAR2, but not ADAR3, deaminate adenosines to inosines, which are interpreted as guanosines by the translational and splicing machinery.^5^ ADARs likely have non-editing-related functions too, but these are not discussed here.

The deamination of cytosine to uridine is catalyzed by the APOBEC (apolipoprotein B mRNA editing enzyme catalytic polypeptide-like) enzyme family. In mammals, APOBEC1 is the main C-to-U enzyme and, differently from ADARs, it needs an essential co-factor (A1CF or RBM47) and auxiliary proteins for deamination. This protein complex, called the editosome, targets single-stranded RNAs by recognizing an 11-nucleotide AU-rich mooring sequence downstream of the editing site.^9^

RNA editing events may affect RNA localization, structure, stability, and transcript processing of both coding and noncoding RNAs (ncRNAs).^10^ Editing is crucial for cell and tissue homeostasis and a variety of human diseases, including both cardiovascular and neurological disorders, have been linked to its deregulation.^11^^,^^12^^,^^13^^,^^14^^,^^15^ Editing in protein-coding mRNA sequences can lead to non-synonymous changes resulting in amino acid alterations (protein recoding or stop codon introduction) and novel protein isoforms. Although this event is not frequent, it takes place in important mammalian genes, such as those encoding the potassium channel Kv11,^16^ the glutamate receptor subunit GluR2,^17^ and the α3 subunit of GABAA (γ-aminobutyric acid type A) receptor (GABRA3)^18^ and Filamin A.^19^ Most RNA editing events, however, occur in 5′ and 3′ untranslated regions (UTRs) and introns of protein coding genes that harbor *Alu* repeats.^11^^,^^20^^,^^21^^,^^22^ Editing in UTRs may affect gene expression through nuclear retention, RNA degradation, and translational regulation, while editing in intronic regions could influence alternative splicing of edited transcripts by modifying or eliminating splice donor and acceptor sites.^23^

Editing also affects ncRNAs. Pri- and pre-microRNAs are targets of RNA editing, which can suppress their processing by Drosha and/or Dicer and, consequently, the expression levels of the mature microRNAs.^24^^,^^25^ Moreover, editing in mature microRNA seed sequences can influence the recognition of binding sites in target mRNAs, thereby changing the microRNA’s set of target mRNAs, its “targetome.”^12^^,^^26^^,^^27^^,^^28^ In addition, RNA editing has the potential to modify microRNA target binding sites, with significant regulatory and functional implications.^22^^,^^29^ Long non-coding RNA (lncRNA) editing can lead to their nuclear retention and alter their biological function by disrupting their binding sites for DNA and RNA molecules as well as for RNA binding proteins.^30^ Biogenesis of circular RNAs (circRNAs) can also be influenced by editing of the parent sequence.^31^

The regulation of RNA editing events is a complex process, as demonstrated by studies highlighting different regulatory mechanisms. For instance, several studies elucidated the influence of genetic loci in regulating RNA editing events.^32^^,^^33^ In addition, other investigations have shown that RNA binding proteins can play a role in modulating RNA editing.^34^ Furthermore, the majority of crucial editing regulators exert their influence in a cell-type-specific fashion.^26^ These findings underscore the intricate nature of RNA editing regulation and the involvement of various factors in shaping the editing landscape.

**Figure 1:**
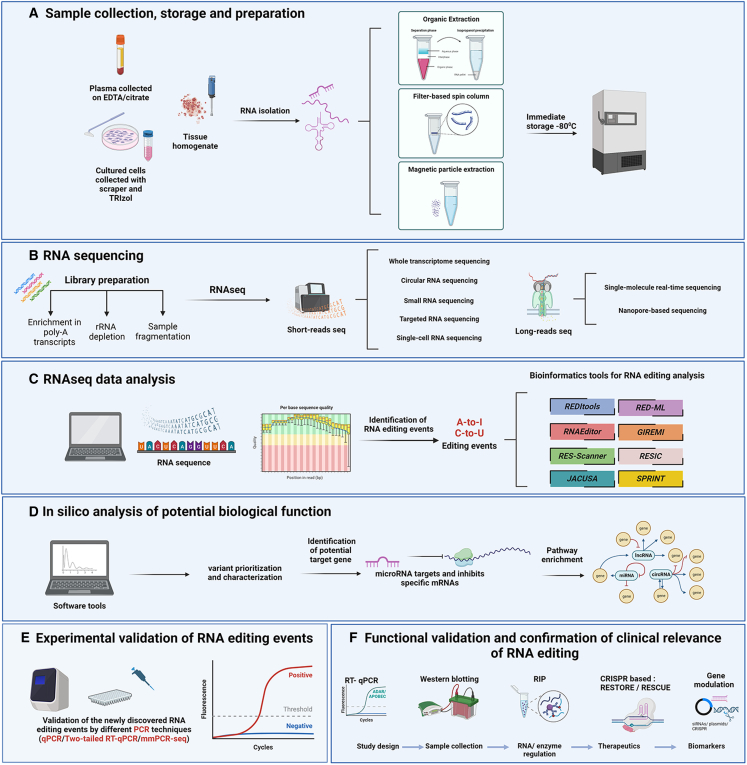
Experimental pipeline for RNA editing studies (A) Summary of the process of sample collection, storage, and RNA isolation, depending on the type of sample. (B) The required steps for RNA-seq library preparation, and the existing RNA-seq approaches depending on the average size and type of RNA. (C) Summary of the main existing bioinformatic tools for RNA editing analysis. (D) Summary of the workflow of utilizing software tools for the identification of potential biological function. (E) Summary of the workflow for experimental validation of RNA editing events, using different PCR techniques. (F) Summary of various experimental methods for the detection of functional effects of RNA editing events, and confirmation of clinical relevance of RNA editing in patient samples. Created with BioRender.com.

RNA editing is clearly a widespread and important mechanism of gene regulation in both health and disease. However, studying RNA editing in biological and clinical settings has its challenges. Therefore, we here provide an experimental guideline for RNA editing studies, using studies from the cardiovascular and neurovascular/neurodegenerative fields as an example, but with applicability to all biomedical research fields. An experimental pipeline, from sample collection to functional and clinical validation, is presented in a single comprehensive figure (Figure 1).

### Sample collection, storage, and preparation

Sample collection, storage, and preparation are of the utmost importance for any RNA sequencing (RNA-seq) experiment, as they dictate its outcome. The protocol for optimal sample collection, independent of RNA editing, is an overall standard procedure but, depending on the type of the sample, there are some differences.

#### Plasma

**Figure 2:**
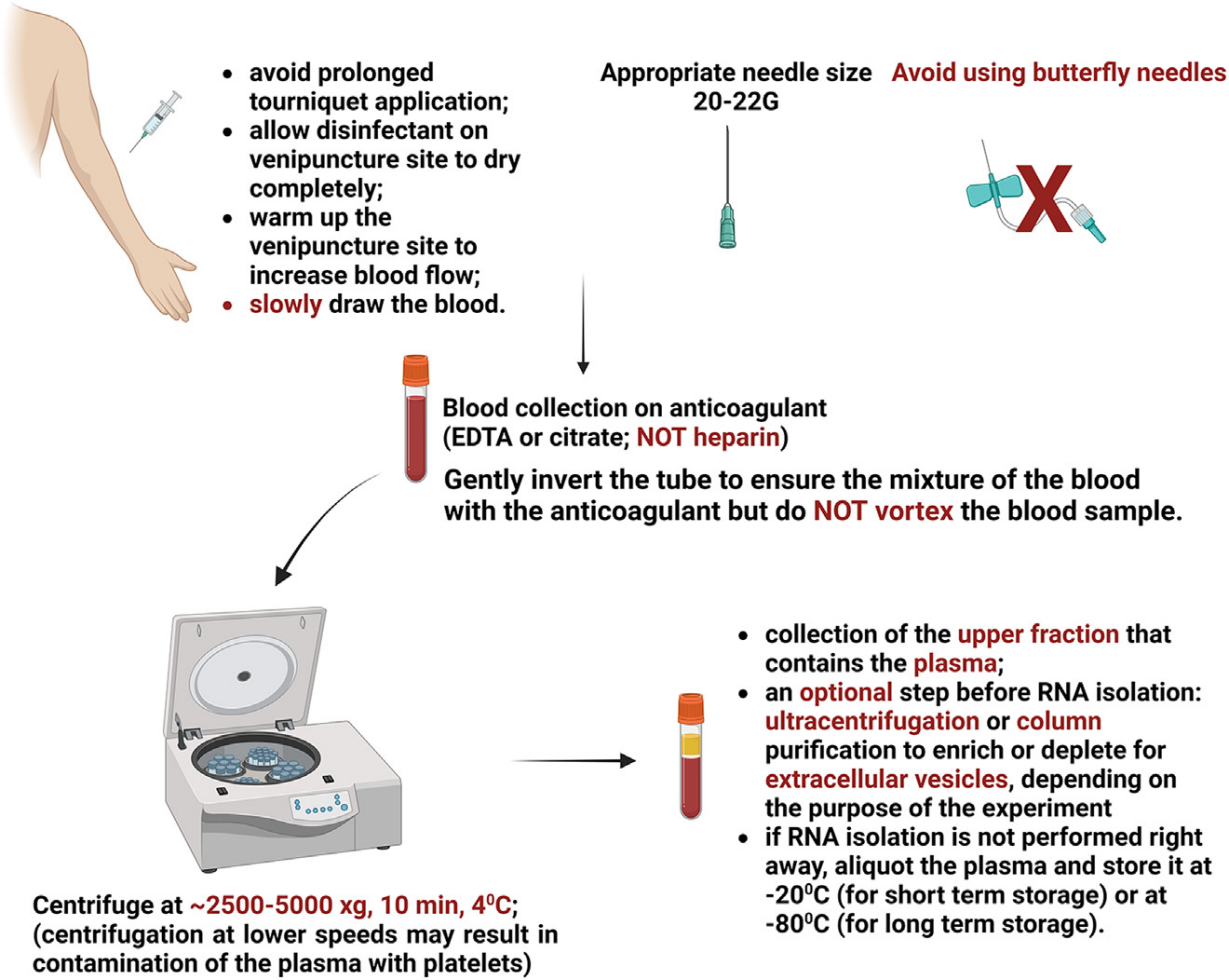
Steps for plasma handling and processing Several crucial steps and pitfalls are highlighted to both warrant high-quality RNA yields from plasma and circulating extracellular vesicles and to avoid hemolysis and platelet contamination. Created with BioRender.com.

Biofluids are intensively studied when looking for new RNA-based biomarkers of various diseases. Among these, plasma, although a great candidate for this as it is readily accessible, is somewhat tricky (Figure 2). One crucial aspect that should be kept in mind when collecting plasma for RNA studies is that heparin should not be used as an anti-coagulant, as it inhibits further PCR assays.^35^ It is recommended that EDTA or citrate tubes be used for plasma collection. If this is not available, heparinase treatment of the RNA isolated from heparin plasma samples is needed before further analysis.^36^ Furthermore, the use of hemolyzed plasma should be avoided for microRNA analysis, as hemolysis can alter the plasma levels of microRNAs that are enriched in red blood cells, which can lead to inaccuracies and misinterpretation of the final results.^37^ In addition, one should consider the use of platelet-poor vs. platelet-rich plasma and avoid unintended platelet activation as platelets carry a large RNA load, including many microRNAs.^38^

#### Cell cultures and tissue samples

The handling of cultured cells or tissues with a view to isolate RNA usually entails the lysis of cells with the help of a cell scraper (for cultured adherent cells) or a tissue homogenizer (for tissues) using either solvents such as TRIzol (Waltham, MA) or other lysis buffers supplied by commercially available kits for RNA isolation. It is important to note that, until recently, due to problems with RNA degradation and formaldehyde-mediated RNA modifications, paraformaldehyde-fixed cells or tissues were not considered as a suitable matrix when RNA-based molecular studies were involved.^39^ This issue has been overcome with a recent high-throughput RNA-seq technique called fixed droplet RNA-seq that was shown to preserve RNA integrity and transcriptomic information in PFA-fixed and permeabilized cell cultures.^40^

It is noteworthy that there are some commercially available solvents, such as DNA/RNA Shield (Zymo Research, Irvine, CA) that can be added to biological samples upon collection to preserve and protect nucleic acid integrity. It is important to consider that the addition of such solvents further dilutes the sample and it might not be suitable for biological samples with a low RNA yield, such as plasma. Cell culture supernatant should be treated in a similar fashion as plasma when it comes to both storage and RNA isolation, given the fact that RNA concentrations are low and extracellular vesicles (EVs) are often present.

#### Sample storage

Storage conditions (time and temperature) of the samples (tissues, cells, or biofluids) during the pre-analytical phase should be strictly controlled to minimize technical variation. Some subtypes of ncRNAs, e.g., microRNAs and circRNAs, are highly stable in different types of samples due to their resistance to degradation.^41^ Nevertheless, since RNA is generally highly unstable, samples should be processed as quickly as possible. When immediate specimen preparation is not feasible, it is recommended to store samples at 4°C or on ice.^42^ For long-term storage, a temperature of −80°C is recommended over −20°C to optimize RNA integrity. The use of commercially available RNA stabilization reagents constitutes an interesting option to preserve RNA during storage and thawing. However, for studies focusing on EVs, the stability of EVs remains an issue and special RNA stabilization reagents are needed. For example, Görgens et al. have shown the use of PBS supplemented with human albumin and trehalose to enhance the stability and improve long-term storage of EV-containing samples at −80°C.^43^ Consistency in the storage conditions is imperative during sample processing, not only to avoid RNA degradation but also to improve the reproducibility of the findings. Consequently, the rigorous documentation of storage information and the inclusion of these data in scientific publications is fundamental.

#### RNA isolation and storage

Obtaining high-quality RNA is also one of the most critical steps for further accurate RNA-seq results. There are three basic RNA isolation approaches with different characteristics and the most suitable one depends on the sample material, the quantity and the type(s) of RNA that needs to be acquired. Organic extraction techniques are widely regarded as the gold standard and they rely on the different solubility of cellular components in solvents such as phenol, ethanol, or isopropanol.^44^^,^^45^ This methodology is ideal for samples high in nucleases (e.g., pancreas) or lipid content (e.g., brain or adipose tissue). Filter-based spin column RNA extraction techniques are based on the absorption of RNA to specific surfaces in the presence of chaotropic salts and is the easiest and safest method available for high-throughput sample processing needs.^46^ Finally, magnetic particle RNA extraction is an easy-to-automate alternative which enables high-throughput procedures.^47^ Until further processing, it is important that RNA be stored in an RNase-free environment, preferably at a temperature of −80°C.

### Different RNA-seq approaches

#### General transcriptome sequencing

Depending on the type of RNAs of interest (coding or noncoding; longer or short), there are some required steps that should be followed when preparing sequencing libraries.

One approach that researchers often use is the selection or enrichment of poly(A) transcripts. In eukaryotes, most mRNAs and many lncRNAs contain a poly(A) tail,^48^^,^^49^ which can be used as a technical tool for selection, either with magnetic or cellulose beads coated with oligo-dT molecules or with oligo-dT priming for reverse transcription (RT).^50^

An alternative approach is the depletion of ribosomal RNA (rRNA), as it is the most abundant RNA type in all cells and it is often of limited interest to most studies. To deplete rRNAs, the one widely used method is the hybridization of rRNA-specific probes, followed by depletion with streptavidin beads.^51^ More specific to ncRNA experiments, the two most widely used methods for rRNA depletion are probe-directed degradation (rRNA targeting by anti-sense DNA oligos and subsequent digestion by RNase H)^52^ and not-so-random priming.^53^ It should be noted that ribodepletion still allows further poly(A) selection of transcripts, so the choice of the technique used should be based on the question or the aim of the conducted research.

The next step consists of sample fragmentation before undergoing RT, which is achieved either by divalent cation treatment with specific enzymes or by using alkaline solutions.^54^ To retain the information pertaining to strand origin, the standard RNA-seq protocol should be modified as follows: during cDNA synthesis, the second-strand synthesis continues as normal except the nucleotide mix includes dUTPs instead of dTTPs; then, after library preparation, a second-strand digestion step is added, which ensures that only the first strand survives the subsequent PCR amplification step and hence the strand information of the libraries.^55^

#### Alternative approaches

RNA-seq alternatives following different approaches should be used depending on the average size of RNAs of interest. Whole-transcriptome approaches sequence both protein coding RNAs (mRNAs) and lncRNAs, due to their structural similarities.^56^ It is most suitable for the discovery of novel transcripts, but some shortcomings are present due to the requirements of large amounts of RNA input and a certain bias due to different sequencing length reads.^57^ Sequencing of circRNAs follows the standard workflow of RNA-seq described above, with an additional step that consists of the digestion of linear RNAs beforehand.^58^

#### Small RNAs

**Figure 3:**
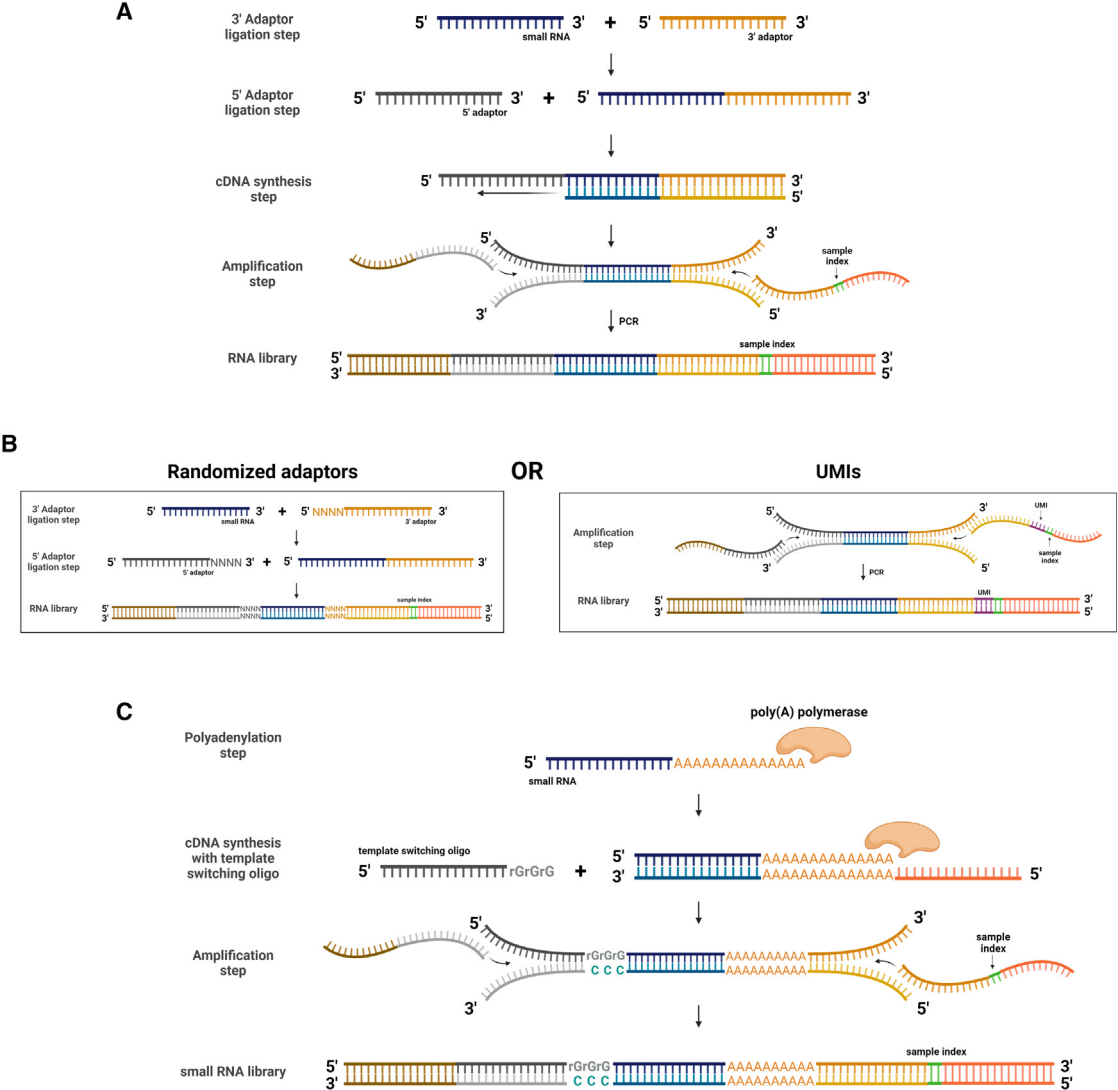
Overview of the main approaches for small RNA-seq analysis (A) Original two-adaptor ligation protocol for small RNA-seq analysis. (B) Improved two-adaptor ligation methods for small RNA-seq analysis. The reduction of PCR or ligation bias can be accomplished through randomized adaptors in the ligation steps or unique molecular identifiers in the amplification step. (C) Ligation-free approach for small RNA-seq analysis through polyadenylation of the 3′ end and template switching method. Created with BioRender.com.

Small ncRNA sequencing, such as microRNA-seq, follows the same principle as lncRNAs, with some modifications. Due to their short size, microRNAs need an extension step either by ligation or by polyadenylation, which may introduce bias,^59^ which can be mitigated by the use of unique molecular identifiers,^60^ which help distinguish reads generated from an identical molecule that was amplified by PCR. Currently, sequencing of small RNAs can be achieved by three main approaches (Figure 3): (1) the original two-adaptor ligation^61^, (2) the improved two-adaptor ligation^62^, and (3) the ligation-free approach.^59^

#### Targeted approaches

Targeted RNA-seq is a specific category that enables selection of specific set of transcripts from defined genes or regions of interest. It is a useful technique for low-expressed transcripts that cannot be easily analyzed through whole-transcriptome sequencing. Until now, the two general approaches commonly used are target capture^63^^,^^64^^,^^65^^,^^66^^,^^67^ and amplicon sequencing.^68^ Target capture is based on the selection of specific regions of interest through hybridization of RNA-seq libraries to a set of biotinylated probes. This method does not require the presence of a poly(A) tail, thus it is ideal for low abundant or degraded RNA. On the contrary, amplicon sequencing utilizes gene-specific primers for the amplification of cDNA targets in combination with a primer for poly(A) tail priming. Target capture seems to provide higher complexity and consistency, while amplicon sequencing has higher on-target rates.^68^

#### mmPCR-seq

The mmPCR-seq method, a large-scale amplicon sequencing technique, has been demonstrated to detect A-to-I editing in both mid-length and longer RNAs.^69^ The method utilizes RT to cDNA followed by amplification of multiple targets in the same pool and subsequent deep sequencing of the resulting products. While amplified sites have been 150–350 base pairs in length in previous studies, it is important to note that target RNAs can be much longer.^69^^,^^70^ The mmPCR-seq method detects RNA editing in the same way as other sequencing methods, through finding A-to-G mismatches to the genome; however, because of the pre-amplification step, mmPCR-seq has been confirmed to precisely measure RNA editing levels in samples even after preamplification of ∼1,000-fold of low-quantity samples.^69^

#### Single-cell sequencing

Finally, single-cell RNA-seq (scRNA-seq) and single-nucleus RNA-seq (snRNA-seq) are two powerful techniques that enable the delineation of transcriptomic cell-to-cell differences, revealing sub-populations with distinct molecular and functional characteristics.^71^ This is especially useful for studies concerning heterogeneous cell populations involved in the development of cardiovascular^72^^,^^73^ and neurodegenerative diseases.^74^^,^^75^ RNA editing studies in mice revealed differences in the editing levels between neurons and glial cells,^76^^,^^77^ while in Drosophila RNA editing levels vary across different neuronal types.^78^ Cell-type-specific RNA editing was also found in the neurons, glutamatergic neurons, medial ganglionic eminence-derived GABAergic neurons, and oligodendrocytes in the human brain.^79^ Challenges concerning single-cell analysis include low inputs of RNA for library construction, difficulty in the detection of low abundant transcripts,^80^^,^^81^ and increased technical and biological noise due to cell-cycle differences.^82^ Since RNA editing analysis requires high sequencing coverage, the detection of RNA editing events in single-cell data is really challenging. Recently, a novel computational approach that attempts to overcome all the aforementioned technical difficulties has been developed.^83^ The novelty of this method is based on the establishment of a pseudo-bulk RNA-seq dataset for each cell type, by integrating the aligned reads of all cells of the same cell type, which significantly increases the sequencing depth and allows the utilization of existing bioinformatics tools for RNA editing detection.

#### RNA modifications

Despite the valuable information that short-read sequencing provides, it is often challenging to capture the true whole diversity and plasticity of all RNA modifications, due to amplification bias (e.g., non-sequence altering modifications are not included in the amplicons) and assembly difficulties.^84^^,^^85^ This is where long-read third-generation sequencing technologies are significant alternatives. These platforms can potentially capture information about assorted modification types simultaneously, in full-length transcripts, at single-molecule resolution, and at a genome- and transcriptome-wide scale.^85^ At the present time, there are two major approaches: (1) single-molecule real-time (SMRT) sequencing technologies^86^ and (2) nanopore-based sequencing technologies.^87^ SMRT sequencing can be used for the detection of several DNA modifications, such as 6-methyladenosine (6mA), 4-methylcytosine (4mC), 5mC, and 5-hydroxymethylcytosine (5hmC). However, the detection of RNA editing and other RNA modifications with SMRT is not yet applicable due to technical restrictions. On the other hand, nanopore sequencing is able to identify both DNA and RNA modifications, not only in naturally occurring alterations but also in artificially induced ones.^85^ Even though nanopore sequencing data for RNA modification detection is still a complex task that presents many challenges,^88^ it seems to be a promising tool for A-to-I editing identification through direct inosine detection.^89^^,^^90^^,^^91^

### Various bioinformatic approaches to the data analysis

There are many challenges in the computational detection of RNA editing events from RNA-seq data, that still need to be addressed, such as the artifacts introduced by high rate of sequencing errors, genomic mutations, the accurate identification of *de novo* editing sites, the tissue-specific editing and the variance in RNA editing percentage in each different editing position.

RNA editing sites can either be detected using RNA-seq data or with a combination of RNA-seq and DNA sequencing (whole-genome sequencing) data from the same sample/individual, to achieve low false positive detection rates due to single-nucleotide polymorphisms (SNPs). When genomic data are unavailable the utilization of SNP annotation databases is crucial. For example, Srivastava et al. demonstrated that RNA editing sites can be predicted with high confidence in mouse brain by eliminating known mouse SNPs.^15^ A critical aspect of RNA editing detection analysis is the sequencing depth and coverage of RNA-seq input. High sequencing depth (80–100 million reads) is essential for accurate and statistically powerful RNA editing site detection.^92^^,^^93^^,^^94^ The inclusion of both biological and technical replicates in the detection pipeline is also an important factor that can increase sensitivity of RNA editing detection.^93^

Various bioinformatic tools and algorithmic choices have been developed in the past few years and depending on the design of the RNA-seq experiment, the available data, and the input parameters the most suitable choice may differ (Table 1).

**Table 1:** Main features of bioinformatic tools for RNA editing detection

Tools	Editing type	Replicates accepted	*De novo* detection of RNA editing	Prior mapping required	Input files	Dependencies	
REDItools Sapiro et al.^78^	both	no	yes	yes	BAM	pysam, BLAT, SAMtools	
RNAEditor Cuddleston et al.^79^	A-to-I	no	yes	no—BWA included	Fastq	pysam, pyqt4, matplotlib, numpy, BWA, Picard Tools, GATK, BLAT, BEDtools	
RES-Scanner Kowalczyk et al.^82^	A-to-I	no	yes	optional—BWA included	Fastq or BAM	Perl modules, BWA, SAMtools, BLAT	
RED-ML Adewale^84^	A-to-I	no	yes	yes	BAM	Perl modules, SAMtools	
GIREMI Lucas and Novoa^85^	A-to-I	yes	yes	yes	BAM	SAMtools, HTSlib, R	
RESIC Light et al.^95^	A-to-I	yes	yes	no	Fastq	SAMtools, Bowtie	
JACUSA Eid et al.^86^	Both	yes	yes	yes	BAM	R for JACUSAhelper	
SPRINT Furlan et al.^88^	Both	no	yes	optional—BWA included	Fastq or BAM	Python, SAMtools, BWA	

REDItools^96^ is the first published software for genome-wide RNA editing site detection and provides both A-to-I and C-to-U variant detection. It is suitable for both RNA-seq and DNA-seq data from the same sample/individual or RNA-seq data alone and uses pre-aligned reads in Binary Alignment/Map (BAM) format as input. It performs *de novo* and known editing site detection and utilizes a wide range of filters and quality control checks for the identification of false positives, especially near intronic splice sites, read-ends and homopolymeric regions. REDItools provides additional scripts for post-processing output tables. These scripts enable the filtering of candidate editing sites based on known annotations, as well as the identification of ambiguous alignments using the tool Blat (BLAST-like alignment tool). Identification of ambiguous alignments is critical to RNA editing site detection.

RNAEditor^97^ provides comprehensive A-to-I variant detection and can be used both from a command line or a graphical user interface. It utilizes RNA-seq data and uses FASTQ files as input that are consequently subjected to a mapping step with BWA aligner.^98^ It can detect both *de novo* and known RNA editing sites and includes a clustering algorithm for “editing islands” detection, through a density-based spatial clustering of applications with noise (DBSCAN).^99^

RES-Scanner^100^ is a fast software package tool for wide identification of RNA editing sites that requires matching RNA-seq and DNA-seq data. FASTQ format or BAM files can be inserted as input for the analysis. RES-Scanner detects both de novo and known editing sites by implementing binomial statistical tests^101^ on possible RNA editing sites, from which the user can adjust the threshold for true positives.

RED-ML^102^ is an efficient RNA editing prediction tool that employs machine learning approaches. Like other tools, RED-ML does not depend on known edited sites and can predict novel edited sites with high accuracy. The tool is designed to process BAM files as input for RNA detection analysis after raw reads alignment. In addition, RED-ML is incredibly fast, making it an ideal choice for researchers looking to predict RNA editing events.

GIREMI^103^ is a software tool for RNA editing prediction that uses RNA-seq data as input, without matching DNA-seq data requirements. Alignment of raw reads is required prior to use, as it only accepts BAM files as input for RNA detection analysis. It can integrate biological replicates into one dataset for higher accuracy or RNA editing detection. GIREMI’s algorithm is based on mutual information between editing sites in RNA-seq data and SNPs, and thus, is suitable only for diploid genomes. Furthermore, a new function for RNA editing detection by long reads has been recently released, applied to PacBio RNA-seq data (L-GIREMI).^104^

RESIC^95^ is a tool for adenosine to inosine RNA editing sites identification and classification based on an alignment graph model and multiple filtering steps. This pipeline can be utilized for all organisms and is suitable for any number of RNA-seq datasets. RESIC enables the detection of RNA editing sites in both repetitive and non-repetitive regions, as well as identify hyper-edited sites. It can also optionally exclude polymorphism sites based on DNA and/or ADAR-mutant RNA-seq datasets. Although this tool provides many functionalities and options, it is not considered user friendly, as it requires manual manipulation of the provided Python script to specify the input data and cannot provide file names as command options.

**Figure 4:**
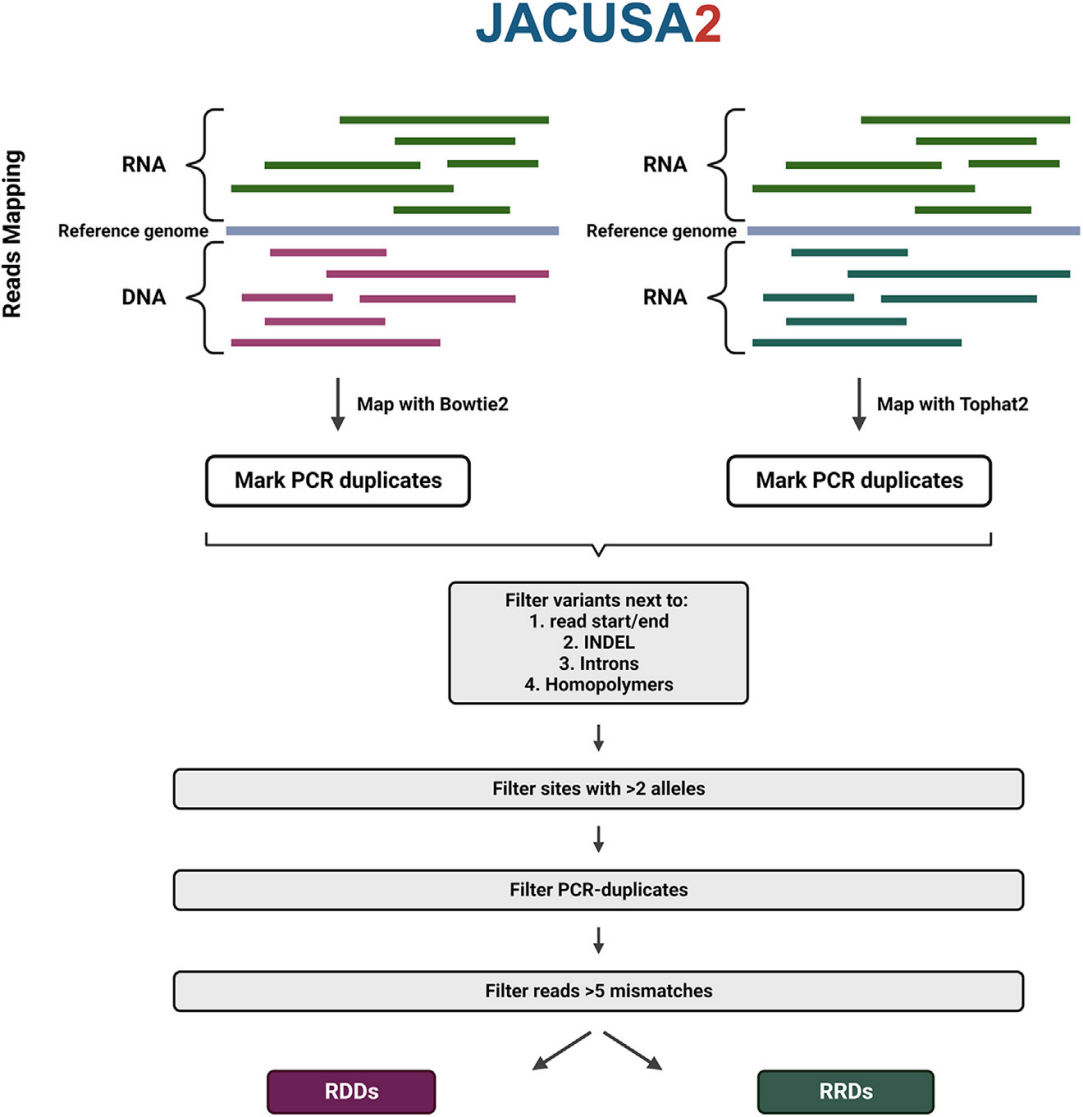
Schematic illustration for JACUSA2 workflow analysis JACUSA2 tool predicts single-nucleotide differences from both RNA-DNA (RDDs) and RNA-RNA (RRDs) sequencing data. PCR duplicates are being marked and variants in the start and end of the genome, in INDELs, in intronic regions and in homopolymers are being filtered accordingly to prevent false-positive RNA editing events. Created with BioRender.com.

JACUSA^105^ detects all RNA nucleotide variants by comparing both RNA-DNA-seq and RNA-RNA-seq data (Figure 4). Unlike any other software tool, it can implement information from multiple experiments, such as biological replicates and different conditions for RNA-RNA differences detection. JACUSA can also integrate information from different library types, such as first- or second-strand cDNA libraries. Recently, an updated tool of JACUSA (JACUSA2) has been released, which presents better time performance and more complex read signatures.^106^

**Figure 5:**
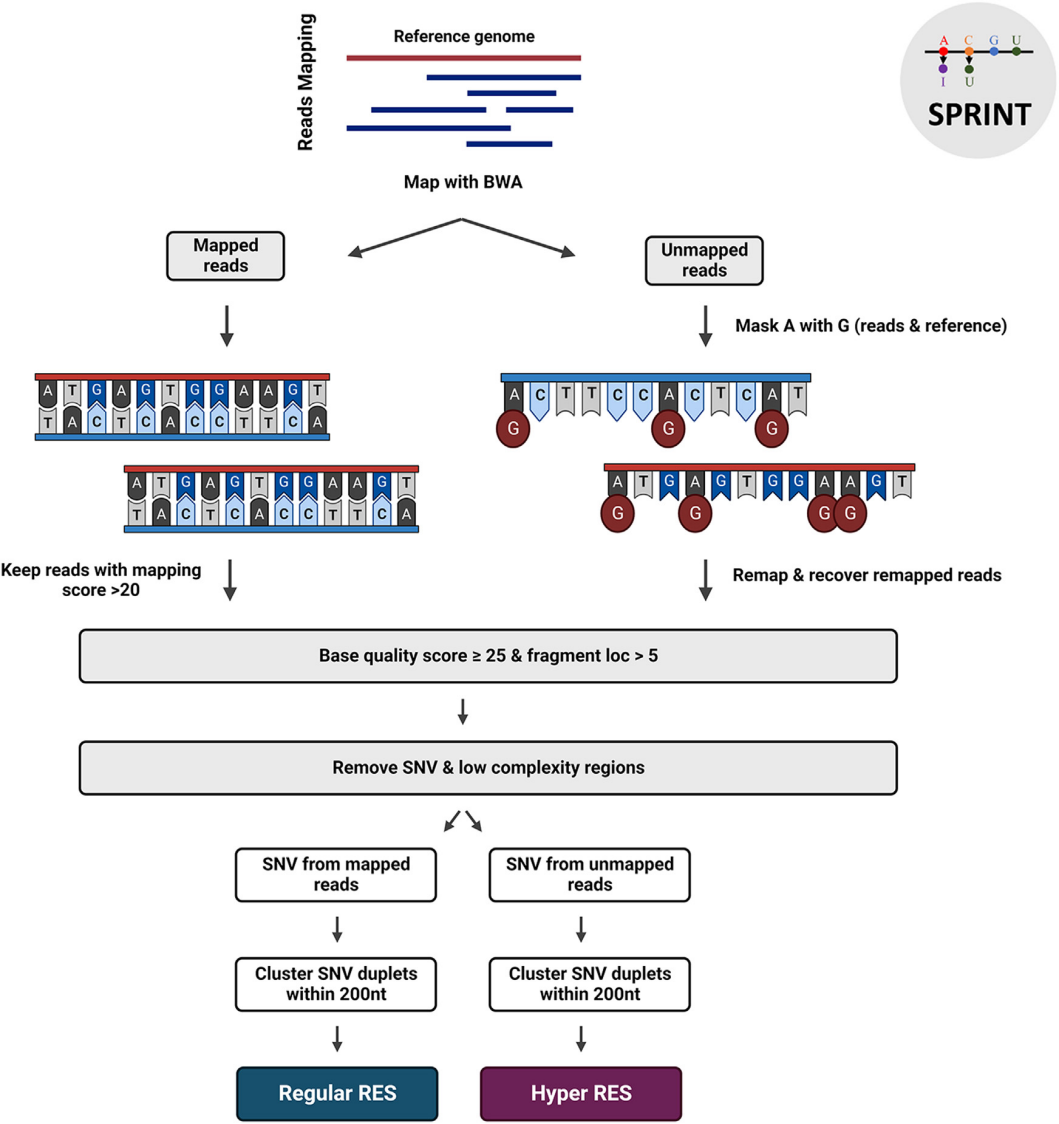
Schematic illustration for SPRINT bioinformatics analysis SPRINT enables the detection of A-to-I and C-to-U editing sites by mapping reads to the corresponding reference genome using Burrows-Wheeler aligner and clustering RES-based and SNP-based SNV duplets. Both regular RES from mapped reads and hyper RES from recovered remapped reads can be identified. Created with BioRender.com.

Finally, SPRINT^107^ is an SNP-free package toolkit for comprehensive A-to-I and C-to-U RNA editing sites detection (Figure 5). The novelty of this tool is that it can identify RNA editing sites (RES) without the need to utilize SNP databases, by clustering RES-based and SNP-based single-nucleotide variant (SNV) duplets, due to their distinctive and unique distribution. In more detail, A-to-I and C-to-U editing are inescapably being observed in different genomic regions, since ADAR enzymes (responsible for A-to-I editing) act only on double-stranded RNA, while APOBEC enzymes (responsible for C-to-U editing) act only on single-stranded RNA. In addition, it is known that ADARs tend to edit sites in clusters (hyper RNA editing) and are prevalent in intronic regions and Alu repeats,^20^^,^^21^^,^^108^^,^^109^^,^^110^^,^^111^^,^^112^ whereas SNPs present independent distribution of each SNP type and low density in the genome. SPRINT is fully automated to any RNA-seq data with available reference genome and can use both raw FASTQ files or BAM files as input. It can detect both *de novo* and known editing sites and can also integrate the detection of hyper RNA editing sites from remapped reads.

#### Online resources

Besides the aforementioned bioinformatics tools, there are several online resources for the detection of RNA editing sites, such as DREAM (detection of RES associated with miRNAs, http://www.cs.tau.ac.il/∼mirnaed/),^113^ REP (prediction of editing sites and their effect in humans) (http://www.rnaeditplus.net/),^114^ and AIRlINER (assessment of editing sites in non-repetitive regions) (http://alpha.dmi.unict.it/airliner/).^115^

#### Editing databases

Databases that host collections of both predicted and validated editing sites are very useful for data comparison, filtering and validation. Currently, RNA editing events are mostly annotated in four main databases: EDK^116^ (https://ngdc.cncb.ac.cn/edk/), REIA (http://bioinfo-sysu.com/reia/),^117^ REDIportal^118^ (http://srv00.recas.ba.infn.it/atlas/) and TCEA (http://tcea.tmu.edu.tw).^119^ EDK is a manually curated database of RNA editing events in mRNAs, miRNAs, lncRNAs, viruses, and RNA editing enzymes, known from the literature to be associated with human diseases. TCEA and REIA integrate RNA editing events specifically focused on cancer research, while REIA also allows both editing profiling and interactive analyses with cancer-related indices. REDIportal is the largest RNA editing resource containing approximately 16 million A-to-I events derived from 9,642 human RNA-seq samples and about 107,094 A-to-I mouse events from RNA-seq data.

### *In silico* analyses of potential biological function

Rapidly progressing sequencing technology has resulted in an explosion of big data^120^ and a subsequent increase in the detection of RNA editing events.^23^ Computational analyses provide mechanistic insights into the large amount of data produced in high-throughput studies. Depending on the different biological questions and produced data, there is a wide variety of approached methods, as well as databases to use.

RNA editing detection follows data assessment and RNA editing sites characterization and validation, all critical and necessary steps for potential connection with a molecular function. RNA editing events can result in codon changes, altered structure, stability, and transcript processing.^13^^,^^23^ Moreover, RNA editing sites can significantly impact the secondary structures of primary/precursor microRNAs and lncRNAs and their overall molecular interaction patterns.^121^

#### Useful software tools

Several software tools are available and support variant prioritization and characterization.^122^^,^^123^^,^^124^^,^^125^^,^^126^^,^^127^^,^^128^^,^^129^ ANNOVAR is an open-source command-line software tool (http://www.openbioinformatics.org/annovar/) that uses pre-compiled annotation databases to annotate SNVs from diverse genomes. Variant effect predictor (VEP) (https://www.ensembl.org/Tools/VEP) is also a well-known and powerful software tool that can be used to analyze data from any species that has an assembled genome sequence and an annotated gene set.^130^ This tool includes a wide range of reference data, including regulatory regions, clinical significance information and biophysical consequences of variants. VEP is available as a command-line software, but also as a user-friendly online tool. Open Custom Ranked Analysis of Variants Toolkit (OpenCRAVAT) (https://opencravat.org/index.html) is a newly developed open-source software tool, combining a wide variety of diverse data resources and computational prediction methods. OpenCRAVAT can be utilized for the annotation of human genetic variation as well as for variant and gene prioritization.^129^ It is available both as a command-line and graphical user interface and is suitable only for human analysis.

#### Pathway enrichment

Pathway enrichment analysis is an important part of RNA editome studies as it provides potential mechanistic insights and biological interpretation of the data. There are many available databases and tools with a great number of pathway collections and interaction networks.^131^^,^^132^^,^^133^^,^^134^^,^^135^^,^^136^^,^^137^^,^^138^ Depending on the platform, there is a variety of structures, pathway content information and visual formats. To name a few, Metascape (https://metascape.org/gp/index.html#/main/step1) is a web-based portal design and provides a comprehensive and up-to-date list of annotation information and analysis resources from 40 independent knowledgebases.^139^ EnrichR (https://maayanlab.cloud/Enrichr/) is also a popular and comprehensive gene set enrichment analysis web server, including more than 200 libraries and over 400,000 terms.^133^^,^^140^ It can integrate the knowledge from many well-regarded projects and provide synthesized information about genes and gene sets. It also offers visualization of the results through bar graphs, cluster-grams, scatterplots, volcano plots and other.

#### ncRNA interactions

A unique category of *in silico* analyses in RNA editome data includes functional analysis of microRNAs and lncRNAs based on their targets, as the majority of the RNA editing sites have unknown functions and exist in noncoding regions of the genome.^141^ There are several resources that store information about microRNAs, such as miRbase, which is one of the main databases that includes a complete microRNA catalogue,^142^ DIANA-TarBase that contains experimentally verified microRNA to gene interactions,^143^ DIANA-miRGen which is a database of microRNA transcriptional regulation,^144^ miRCarta^145^ and mirGeneDB.^146^ There are also several microRNA target prediction tools based on sequence complementarity. DIANA-microT-CDS is a web server hosting *in silico* predictions of microRNA-mRNA interactions.^147^ miRWalk is an open-source platform that can generate both predicted and validated miRNA-binding sites of known genes in human, mouse and other model organisms.^148^ DIANA-miRPath is a tool that enables the functional annotation of a microRNA or the combined effect of multiple microRNAs, as well as the identification of microRNA-controlled pathways.^149^ DIANA-mirExTra allows the identification of microRNAs that control mRNAs, transcription factors (TFs) regulating mRNAs and TFs regulating microRNAs between two conditions.^150^ TargetScan is an online prediction tool model that utilizes the presence of conserved 8mer, 7mer, and 6mer sites for the detection of biological targets of miRNAs.^151^ Another popular algorithm is TarPmiR, which enables the prediction of microRNA target sites using four different machine learning methods to CLASH (crossinking, ligation and sequencing of hybrids) data.^152^ Finally, there are also many databases that host information about lncRNAs. LNCipedia^153^ and lncRNome^154^ provide numerous human lncRNA entries including primary sequences and predicted secondary structures. DIANA-LncBase is the first database to include experimentally supported information about microRNA-lncRNA interactions.^155^ LNCediting is a user-friendly database providing customized tools to predict functional effects of novel editing.^121^

### Experimental validation of RNA editing events

**Figure 6:**
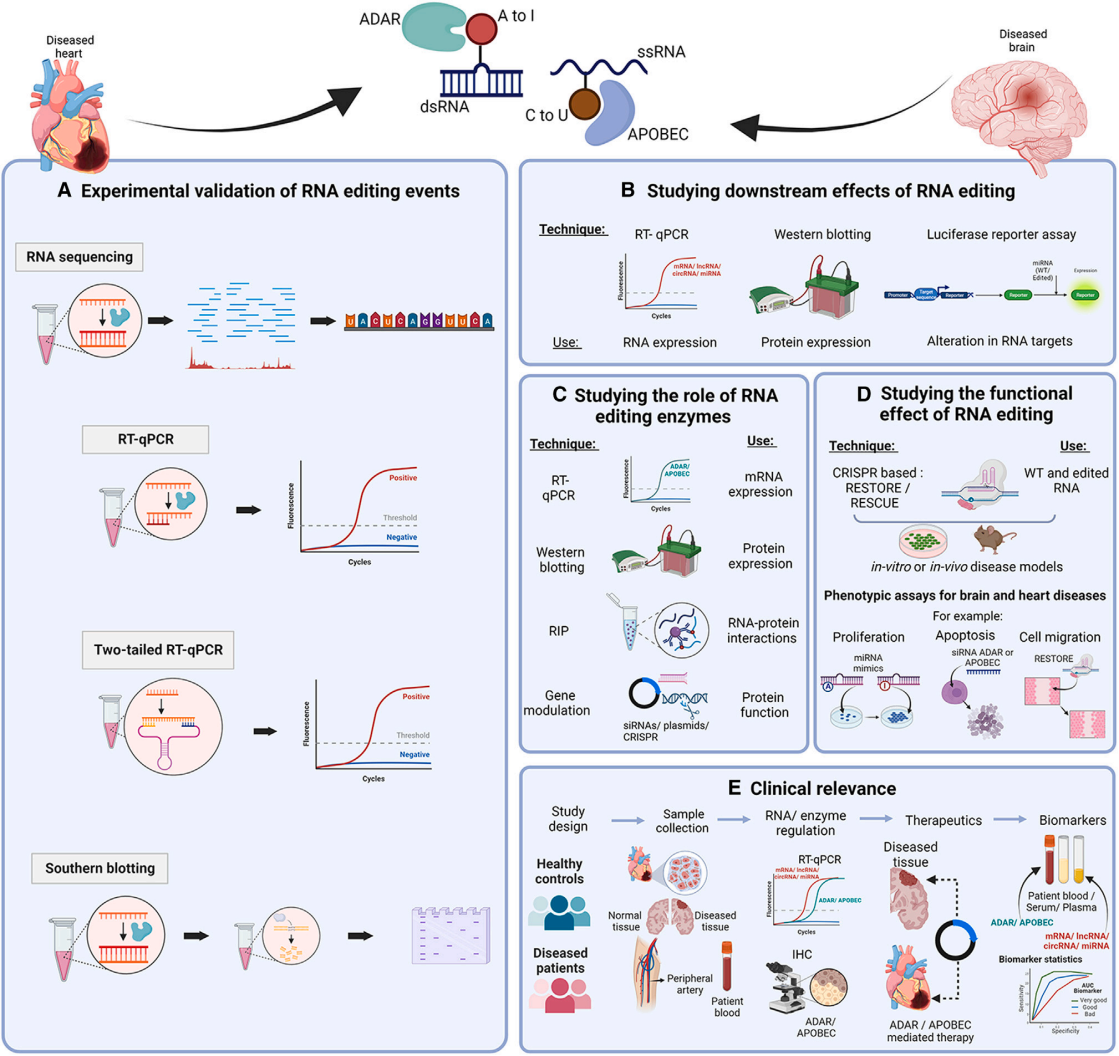
Experimental validation and clinical relevance of RNA editing events (A) Various methods to discover, detect, validate, and quantify individual RNA editing sites. Top to bottom, the panel shows RNA-seq, allele-specific RT-qPCR, two-tailed RT-qPCR, mmPCR sequencing, and restriction fragment-length polymorphism analysis of cDNA by southern blotting. (B) RT-qPCR to help in studying the impact of RNA editing on the expression of parent RNAs (mRNAs, lncRNAs, circRNAs, or miRNAs) or their downstream targets. Western blotting helps to study the effect of RNA editing on the protein expression of edited parent coding RNA or its downstream protein target/s. Luciferase reporter gene assays help in validate any alterations in the targetome of edited RNAs. (C) Methods to study the role of RNA editing enzymes. From top to bottom the panel shows RT-qPCR, western blotting, RNA binding protein immunoprecipitation (RIP), and various methods for DNA/RNA modulation. (D) Methods to study the functional effect of RNA editing events. CRISPR-based techniques such as RESTORE and RESCUE can be used to generate unedited and edited RNA molecules for *in vitro* or *in vivo* studies. Phenotypic assays such as proliferation assays, apoptosis assay, or cell migration assay help to identify the functional role of RNA editing in disease models. (E) Focus on clinical relevance. Clinical studies collecting patient and control blood samples or tissue biopsies can be used to verify association between RNA editing and disease or between RNA editing and drug (in-)sensitivity, for example. Furthermore, edited RNAs or editing enzymes may serve as novel disease-specific biomarkers. Created with BioRender.com.

As described above, new RNA editing events can be found using RNA-seq. However, the higher cost and complexity of processing samples and analyzing the data are significant disadvantages that can limit the practical use of RNA-seq in certain settings. High-throughput sequencing, e.g., next-generation sequencing allows sequencing millions of RNA fragments simultaneously and it is used to detect RNA editing events genome-wide.^156^ Sequence data is then aligned to the reference genome. However, infrequently read sites may be incorrectly labeled as a misread or short reads may be aligned incorrectly.^156^ Validation of individual editing events by different methods is therefore necessary (Figure 6A).

#### RT-qPCR

RT-qPCR with custom primers is a widely used method to analyze relative expression of different RNAs.^157^ However, its ability to properly distinguish single-nucleotide changes must be confirmed for each individual editing event.^26^^,^^158^ Particularly for small RNAs such as microRNAs a problem in distinguishing microRNA isoforms arises, because they often differ by only one nucleotide, and closely related isoforms may produce background signals in qPCR due to mismatches in primer annealing. Novel methods for validation of suspected RNA editing sites have been developed to overcome this problem, such as two-tailed RT-qPCR^157^ and microfluidics-based multiplex PCR and deep sequencing (mmPCR-seq).^69^

Two-tailed RT-qPCR can be used to reliably quantify A-to-I modifications of microRNAs.^157^^,^^158^ It utilizes two short, approximately 5–10 nucleotides in length, hemiprobes, connected by a hairpin structure. The target edited nucleotide is designed inside the short primer, and a single-nucleotide mismatch has been confirmed to disturb the pairing of the primer and the target RNA, making it possible to distinguish even single-nucleotide differences.

#### Southern blotting

An alternative method for detecting both A-to-I and C-to-U RNA editing in mid-length to long RNAs is analyzing cDNA fragments by Southern blotting/electrophoresis after digestion by sequence-specific restriction enzymes to reverse-transcribed and PCR-amplified cDNA.^27^^,^^159^ The objective of the electrophoresis method is cleaving the wild-type isoform using a specific restriction enzyme, while RNA editing prevents the enzyme cleaving the isoform, or vice versa. Importantly, synthetic cDNA is used to confirm both specificity and efficacy of the restriction enzyme, and genomic DNA is used to confirm the absence of an A-to-G SNP (van der Kwast et al.^27^; supplemental information). Computer-assisted densitometric analysis can be used to objectively measure the levels of different isoforms.^27^ Alternatively, undigested amplified cDNA can also be analyzed by Sanger sequencing and compared with genomic DNA sequences.^27^

### Functional validation and confirmation of clinical relevance of RNA editing

RNA editing can affect both expression and function of all species of RNAs. Therefore, in this part of the article, we discuss methods to functionally validate as well as evaluate the clinical impact of RNA editing, using studies from the cardiovascular and neurological fields as examples.

To functionally validate the impact of RNA editing, the effect on its parent RNA expression, its resulting protein (for mRNAs), and/or its downstream targets (for both coding and ncRNAs) must be analyzed^26^^,^^160^ (Figure 6B).

#### RNA expression

Potential effects of editing on RNA expression can be measured by RT-qPCR assays designed for both protein-coding and ncRNAs. As an illustration, Paul et al. used qPCR to demonstrate that four distinct hypo-edited microRNAs were downregulated in brain samples of patients with glioblastoma multiforme (GBM) in contrast to corpus callosum samples obtained from non-GBM deceased patients, confirming effects of RNA editing on the expression of the mature microRNAs.^161^

#### Protein expression

To determine modulations in protein expression caused by RNA editing of mRNAs, classical western blotting or immunohisto/cytochemistry can be employed, such as done by Jain et al., who looked at the effects of an RNA editing-directed amino acid change on the protein expression of Filamin A using western blotting. The authors showed that the amino acid change (Q-to-R), caused by a highly conserved A-to-I RNA editing event, can lead to a decrease of Filamin A protein expression in primary mouse lung fibroblasts.^162^

To validate indirect effects of RNA editing on gene expression, luciferase reporter gene assays are commonly used. For example, increased editing of a specific subset of microRNAs under ischemia alters the microRNAs’ targetomes, as demonstrated by van der Kwast et al., who cloned target mRNA 3′ UTR sequences with single or multiple microRNA binding sites (unedited vs. edited) into the PsiCheck2 luciferase reporter gene vector. Co-transfection of the vector with either unedited or edited microRNA mimics in HeLa cells resulted in significant changes in luciferase activity, confirming effects on target binding and gene expression.^26^^,^^27^

#### ADARs and APOBECs

The genes encoding the ADAR and APOBEC families of deaminases can produce several variants or isoforms of these enzymes. Thus, it is important to characterize the enzymes and determine which family member(s) and isoform(s) are responsible for the observed RNA editing patterns (Figure 6C). To accomplish this, RT-qPCR at mRNA level and western blotting or immunohisto/cytochemistry at protein level can detect changes in expression of these enzymes.^26^^,^^27^^,^^160^^,^^161^^,^^163^^,^^164^ In a study by Kokot et al., investigating failing human hearts, both ADAR1 and ADAR2 were examined. While qPCR did not show a regulation at transcriptional level, western blotting showed that both ADAR1 and ADAR2 were regulated in heart failure samples compared with samples from non-failing hearts.^163^

Besides enzyme expression, direct interaction of the deaminases with the edited RNA should be analyzed. An effective way to achieve this is by using RNA immunoprecipitation (RIP) with an antibody specific to the deaminase in question. Many protocols relating to RIP to identify targets for RNA-binding proteins (RBPs) have been published, which mainly include RIP-seq, RIP-ChIP-seq, and RIP followed by RT-qPCR. To study the effect of ADARs on RNA editing, RIP-seq for high-throughput analysis for target RNA identification can be performed.^165^ A similar screening approach, using high-throughput RNA-seq after ADAR1-RIP, showed the interaction of ADAR1 with highly edited lncRNAs.^10^ Furthermore, to study a specific RNA transcript as target, RIP followed by RT-qPCR could be used. RIP-RT-qPCR involves immunoprecipitation of the RBP and performing targeted RT-qPCR on the co-precipitated RNA.^165^ For instance, in HEK293 cells, ADAR2-specific RIP confirmed its interaction with a pre-mRNA AKAP13, leading to reduced AKAP13 RNA stability and circularization in failing human hearts.^163^

To further study the downstream effects of the deaminases, knockin and knockdown experiments using plasmids, siRNAs, viral vectors, or CRISPR-based techniques can be used to mimic effects of ADAR or APOBEC modulation on RNA editing events. For example, an siRNA against ADAR1 or ADAR2 in human umbilical cord fibroblast cells was used to determine the effects of ADAR1 and/or ADAR2 expression on editing of both primary and mature microRNAs under ischemic conditions.^26^ Even though siRNAs have been shown to effectively silence ADARs, the off-target effects of silencing such as modulation of editing patterns in other genes or changes in gene expression via miRNA processing should be noted. In this regard, other approaches might be useful. ADAR2, for example, besides its active site, binds to its target RNA via several residues present in three main regions close to the RNA editing sites.^166^ As such mutating the specific residues could help in targeted mimicking of RNA editing, thereby reducing the off-target effects. A similar approach can be applied to modulate APOBEC expression to study editing of both coding and ncRNAs.^156^^,^^167^ Thus, studying the role of deaminases in the regulation of edited coding or ncRNAs can provide a foundation for further functional studies.

#### Experimental induction of RNA editing

For functional analysis, it is crucial to develop methods for generating the unedited and edited versions of the target RNA Figure 6D). Recently, efficient CRISPR-based tools have been developed to achieve this, which involve recruitment of the endogenous editing enzymes to specific sites in RNAs, using guide RNAs, to create edited transcripts. An example for A-to-I editing is RESTORE (recruiting endogenous ADAR to specific transcripts for oligonucleotide-mediated RNA editing).^168^ RESTORE involves engineering of anti-sense oligonucleotides (ASOs) containing chemically active ADAR recruiting domains. Upon transfection into different human cell types, these ASOs could recruit endogenous ADAR, causing editing of endogenous transcripts while maintaining the natural editing homeostasis.^168^ Another system, using ADAR1/2 enzymes along with ADAR specific guide RNAs, has been developed for targeted A-to-I RNA editing in both *in vitro* and *in vivo* models.^169^

Another RNA editing method called LEAPER (leveraging endogenous ADAR for programmable editing of RNA) uses engineered ADAR recruiting RNAs (arRNAs) to recruit native ADAR1 or ADAR2 to facilitate A-to-I transition on target RNAs. Recently, an updated version of LEAPER, called LEAPER 2.0 has been described. LEAPER 2.0 uses circular ADAR recruiting RNAs (circ-arRNAs) to achieve precise RNA editing with higher efficiency and less off-target effects in both *in vitro* and *in vivo* conditions.^170^

Jain et al. generated both fully unedited and fully edited Filamin A mice, leading to a Q-to-R amino acid change. These mice were then used to functionally validate the role of Filamin A editing in both tumor and ischemia models.^171^ For C-to-U editing, Bhakta et al. engineered an RNA editing enzyme complex by using an MS2-tagged system and a combination of the deaminase domain of APOBEC1 with a guide RNA, complementary to the blue fluorescence protein mRNA, creating a C-to-U point mutation, which resulted in a green fluorescence protein.^159^

These model systems with unedited and edited RNAs can be used to investigate the role of RNA editing in disease progression in multiple disease phenotypes. For example, using 3D spheroid assays, proliferation assays, transwell cell migration assays, and hindlimb ischemia assays, Jain et al. demonstrated the role of Filamin A mRNA editing in angiogenesis, tumor growth, metastasis, and post-ischemic blood flow recovery, respectively, in a murine hindlimb ischemia model.^171^

Focusing on C-to-U editing, knockout of APOBEC1 was shown to be associated to severity of experimental autoimmune encephalomyelitis, a mouse model mimicking multiple sclerosis. Using techniques such as hematoxylin and eosin staining and immunostaining, Dafou et al. demonstrated the role of APOBEC1 in causing increased inflammation, gliosis, astrocytosis, and T cell infiltration in the brain of APOBEC1 knockout mice presenting the role of C-to-U RNA editing in neurological diseases.^167^ As such, functional assays appropriately designed for the disease type can provide additional evidence on the impact of editing of diverse RNA species on disease phenotype.

A more direct approach is feasible when studying small RNAs. In two different studies, van der Kwast et al. looked at vasoactive microRNAs that are edited under ischemia. MicroRNAs of the 14q32 gene locus play a directing role in vascular remodeling and in the adaptive vascular response to ischemia. In their studies, van der Kwast et al. show that ischemia induces editing of several 14q32 microRNAs, which alters their targetome and leads to an enhanced pro-angiogenic activity. Using commercially available microRNA mimics, the authors induced overexpressing of either the unedited or edited microRNAs. Using different functional assays, the authors demonstrated that the unedited microRNAs inhibit angiogenesis, whereas the edited microRNAs induce angiogenesis, both *in vitro* and *ex vivo*.^26^^,^^27^

#### Clinical relevance

Dysregulation of RNA editing has been implicated in several diseases including cardiovascular and neurological diseases.^11^^,^^172^^,^^173^ An effective way to establish clinical impact is to study global editing events and their influence on gene expression in patient samples (Figure 6E).

In the cardiovascular disease context, using *ex vivo* cultured human arteries and veins, van der Kwast et al. showed an association of microRNA editing with peripheral artery disease, by demonstrating active regulation of microRNA editing during ischemia.^26^ In a study that aimed to delineate the role of ADARs in congenital heart disease, Altaf et al. found a downregulation of ADAR2 in 35 whole-blood samples and left ventricular tissues of patients with dilated cardiomyopathy compared with 13 healthy controls, suggesting a role of ADARs in cardiovascular disease pathogenesis.^174^ Considering these findings, RNA editing enzymes such as ADARs may be promising biomarkers in cardiovascular diseases, as is further discussed below.

In the neurological/neurodegenerative disease context, decreased levels of global A-to-I editing were observed in postmortem brain tissues of 55 patients with Alzheimer’s disease, compared with 44 non-demented controls.^172^ Besides Alzheimer’s, editing is associated with Creutzfeldt-Jakob disease and other neurodegenerative disorders.^13^^,^^175^^,^^176^ Furthermore, multiple studies have shown decreased ADAR2-mediated RNA editing in the motor neurons of patients with amyotrophic lateral sclerosis (ALS). In this regard, restoring ADAR2 activity and targeting dysregulated RNA editing may be a promising novel approach for future ALS therapy.^177^

Besides developing therapeutics directed at editing enzymes or at specific editing events, it has been shown that RNA editing can influence sensitivity to certain drugs, such as a group of synthetic compounds known to act as open-channel blockers that target voltage-activated potassium (Kv) channels in neurons.^178^ Via such mechanisms, RNA editing may offer a challenge in (future) drug development but may also offer opportunities toward personalized medicine. As such, along with the development of RNA editing drugs, it is imperative to define their safety and effectiveness in the specific cell types where they are intended to be used. Moreover, innovative approaches to guide the drugs to the cell type of interest and methods to follow the effects of these drugs are needed.

#### Biomarkers

As briefly mentioned above, ADAR2 expression, but potentially also editing events themselves, may have potential as disease biomarkers. To determine the validity of a biomarker, a commonly used statistical test is the area under the curve (AUC) of a receiver operating characteristic plot. An AUC of >0.8 indicates a good diagnostic discrimination.^179^ In a recent study, Salvetat et al. showed that whole-blood RNA editing variant detection as potential biomarker for diagnosing patients with depression (n = 267) compared with controls (n = 143) has an AUC of 0.930. In addition, a combination of six RNA editing related biomarkers were able to differentially diagnose patients with unipolar (n = 160) vs. bipolar disorder (n = 95; AUC = 0.935).^180^

### Impact and translational/therapeutic perspectives

RNA editing has been revealed as an additional level of complexity in gene regulation and dysregulation. This regulation affects both short and long RNAs, protein-coding RNAs, and ncRNAs. Changes in nucleotide sequences have been shown to be regulated in several pathological conditions, hence constituting an interesting and relatively novel field of investigation. Discovery of associations between changes in RNA editing and disease development and progression has the capacity to bring not only an enhanced knowledge of the molecular mechanisms beyond these associations, but also to unravel novel therapeutic targets and biomarkers. Indeed, with the rapid evolution of RNA-seq techniques, the possibility to “repair” genes or RNA transcripts using CRISPR-based technology, or the feasibility to measure circulating levels of edited RNA transcripts, RNA editing studies may well lead to the testing of novel treatments and diagnostic strategies. These strategies can be applied to any disease, not only brain and heart diseases, which have been taken as examples in the present article. In favor of RNA editing as a therapeutic tool, *in vivo* preclinical studies using ADAR-mediated RNA editing to correct missense and nonsense mutations have been reported. In two Rett syndrome mouse models, ADAR was used to repair the *Mecp2* mutation and protein function.^181^ In another mouse study, cardiac-specific ADAR2 overexpression protected against doxorubicin-induced cardiotoxicity and decreased cardiac injury and fibrosis upon acute myocardial infarction, suggesting that ADAR editing may be a promising therapeutic strategy for heart diseases.^182^ Mechanistically, ADAR2 stimulated neonatal cardiomyocyte proliferation and inhibited doxorubicin-induced cardiotoxicity by affecting the maturation of miR-34a primary transcript and regulating the expression of its target genes Sirt1, Cyclin D1, and Bcl2.

Several Biotech and Pharma companies aim to leverage RNA editing for novel therapeutic strategies, in the brain and heart fields, but not only in these fields. It is expected that these companies will allow translating research findings to clinical application. However, many challenges remain to be solved before applying RNA editing as novel therapeutics, such as specificity, dosage, safety, and so on. Besides the “common” challenges that affect all novel therapeutic strategies, an additional, and perhaps hard to overcome, challenge for therapies that target RNA editing may be the extreme spatiotemporal specificity of both RNA expression and RNA editing. When looking at microRNAs, for example, we know that their expression levels can vary greatly within the same organ or tissue,^183^ that their target mRNAs are different from cell type to cell type within a single organ or tissue,^184^ and also that editing levels of the exact same editing site can vary greatly between different cell types within the same tissue.^26^^,^^27^ This implies that an extremely detailed understanding of spatiotemporal RNA editing is required for any editing-based therapeutic strategy to be both safe and effective, underlining the importance of the complex methodology discussed above.

As for any novel field of investigation, the research on RNA editing has a strong potential to generate intellectual property rights, innovative drugs, and molecular diagnostic tests. Care must be taken when designing research projects, to make sure that the developed tools are clinically translatable. Involving end-users in research projects, from their inception (grant application level) to their development and finalization, is crucial to ensure adoption of developed tools and translation to the clinic. Not only clinicians and private commercialization partners, but also patient organizations should be involved in every step of a project, from its very early inception/design phase, to its implementation or testing phase by end-users, which are patients overall (https://patient-engagement.eu/). Engaging patient organizations as full partners in research projects ensures a higher level of translatability, and adoption of novel solutions, for the individual patient’s benefit. Especially with mechanisms such as RNA editing, that has been linked to very tight, tissue-specific regulation and to individual drug (in)sensitivity, the potential for both biomarkers and personalized medicine seems very high.

## Conclusions

Even though there are still considerable challenges in RNA editing research, we present a complete study and analysis pipeline here that enables RNA editing studies in biological samples all the way from RNA editing site discovery, through validation and functional analysis, to clinical relevance. The examples used here, taken from the cardiovascular and neurological/neurodegenerative disease fields, underline the importance of RNA editing research. Novel tools are continuously being developed that will facilitate future RNA editing studies even more.

It should be noted that RNA editing comprises just 2 of over 170 different types of RNA modifications that have been discovered to date. Therefore, we look forward to a great number of highly exciting RNA modification studies in the near future.

## References

[1] Benne R., Van den Burg J., Brakenhoff J.P., Sloof P., Van Boom J.H., Tromp M.C. (1986). Major transcript of the frameshifted coxII gene from trypanosome mitochondria contains four nucleotides that are not encoded in the DNA. Cell.

[2] Wagner R.W., Smith J.E., Cooperman B.S., Nishikura K. (1989). A double-stranded RNA unwinding activity introduces structural alterations by means of adenosine to inosine conversions in mammalian cells and Xenopus eggs. Proc. Natl. Acad. Sci. USA.

[3] Powell L.M., Wallis S.C., Pease R.J., Edwards Y.H., Knott T.J., Scott J. (1987). A novel form of tissue-specific RNA processing produces apolipoprotein-B48 in intestine. Cell.

[4] Chen S.H., Habib G., Yang C.Y., Gu Z.W., Lee B.R., Weng S.A., Silberman S.R., Cai S.J., Deslypere J.P., Rosseneu M. (1987). Apolipoprotein B-48 is the product of a messenger RNA with an organ-specific in-frame stop codon. Science.

[5] Nishikura K. (2016). A-to-I editing of coding and non-coding RNAs by ADARs. Nat. Rev. Mol. Cell Biol..

[6] Melcher T., Maas S., Herb A., Sprengel R., Seeburg P.H., Higuchi M. (1996). A mammalian RNA editing enzyme. Nature.

[7] Tan M.H., Li Q., Shanmugam R., Piskol R., Kohler J., Young A.N., Liu K.I., Zhang R., Ramaswami G., Ariyoshi K. (2017). Dynamic landscape and regulation of RNA editing in mammals. Nature.

[8] Raghava Kurup R., Oakes E.K., Manning A.C., Mukherjee P., Vadlamani P., Hundley H.A. (2022). RNA binding by ADAR3 inhibits adenosine-to-inosine editing and promotes expression of immune response protein MAVS. J. Biol. Chem..

[9] Fossat N., Tourle K., Radziewic T., Barratt K., Liebhold D., Studdert J.B., Power M., Jones V., Loebel D.A.F., Tam P.P.L. (2014). C to U RNA editing mediated by APOBEC1 requires RNA-binding protein RBM47. EMBO Rep..

[10] Wang I.X., So E., Devlin J.L., Zhao Y., Wu M., Cheung V.G. (2013). ADAR regulates RNA editing, transcript stability, and gene expression. Cell Rep..

[11] Stellos K., Gatsiou A., Stamatelopoulos K., Perisic Matic L., John D., Lunella F.F., Jaé N., Rossbach O., Amrhein C., Sigala F. (2016). Adenosine-to-inosine RNA editing controls cathepsin S expression in atherosclerosis by enabling HuR-mediated post-transcriptional regulation. Nat. Med..

[12] Kawahara Y., Megraw M., Kreider E., Iizasa H., Valente L., Hatzigeorgiou A.G., Nishikura K. (2008). Frequency and fate of microRNA editing in human brain. Nucleic Acids Res..

[13] Karagianni K., Pettas S., Christoforidou G., Kanata E., Bekas N., Xanthopoulos K., Dafou D., Sklaviadis T. (2022). A Systematic Review of Common and Brain-Disease-Specific RNA Editing Alterations Providing Novel Insights into Neurological and Neurodegenerative Disease Manifestations. Biomolecules.

[14] Nossent A.Y. (2022).

[15] Srivastava P.K., Bagnati M., Delahaye-Duriez A., Ko J.H., Rotival M., Langley S.R., Shkura K., Mazzuferi M., Danis B., van Eyll J. (2017). Genome-wide analysis of differential RNA editing in epilepsy. Genome Res..

[16] Streit A.K., Derst C., Wegner S., Heinemann U., Zahn R.K., Decher N. (2011). RNA editing of Kv1.1 channels may account for reduced ictogenic potential of 4-aminopyridine in chronic epileptic rats. Epilepsia.

[17] Higuchi M., Single F.N., Köhler M., Sommer B., Sprengel R., Seeburg P.H. (1993). RNA editing of AMPA receptor subunit GluR-B: a base-paired intron-exon structure determines position and efficiency. Cell.

[18] Daniel C., Wahlstedt H., Ohlson J., Björk P., Ohman M. (2011). Adenosine-to-inosine RNA editing affects trafficking of the gamma-aminobutyric acid type A (GABA(A)) receptor. J. Biol. Chem..

[19] Levanon E.Y., Hallegger M., Kinar Y., Shemesh R., Djinovic-Carugo K., Rechavi G., Jantsch M.F., Eisenberg E. (2005). Evolutionarily conserved human targets of adenosine to inosine RNA editing. Nucleic Acids Res..

[20] Levanon E.Y., Eisenberg E., Yelin R., Nemzer S., Hallegger M., Shemesh R., Fligelman Z.Y., Shoshan A., Pollock S.R., Sztybel D. (2004). Systematic identification of abundant A-to-I editing sites in the human transcriptome. Nat. Biotechnol..

[21] Athanasiadis A., Rich A., Maas S. (2004). Widespread A-to-I RNA editing of Alu-containing mRNAs in the human transcriptome. PLoS Biol..

[22] Gu T., Buaas F.W., Simons A.K., Ackert-Bicknell C.L., Braun R.E., Hibbs M.A. (2012). Canonical A-to-I and C-to-U RNA editing is enriched at 3'UTRs and microRNA target sites in multiple mouse tissues. PLoS One.

[23] Uchida S., Jones S.P. (2018). RNA Editing: Unexplored Opportunities in the Cardiovascular System. Circ. Res..

[24] Yang W., Chendrimada T.P., Wang Q., Higuchi M., Seeburg P.H., Shiekhattar R., Nishikura K. (2006). Modulation of microRNA processing and expression through RNA editing by ADAR deaminases. Nat. Struct. Mol. Biol..

[25] Kawahara Y., Zinshteyn B., Chendrimada T.P., Shiekhattar R., Nishikura K. (2007). RNA editing of the microRNA-151 precursor blocks cleavage by the Dicer-TRBP complex. EMBO Rep..

[26] van der Kwast R.V.C.T., Parma L., van der Bent M.L., van Ingen E., Baganha F., Peters H.A.B., Goossens E.A.C., Simons K.H., Palmen M., de Vries M.R. (2020). Adenosine-to-Inosine Editing of Vasoactive MicroRNAs Alters Their Targetome and Function in Ischemia. Mol. Ther. Nucleic Acids.

[27] van der Kwast R.V.C.T., van Ingen E., Parma L., Peters H.A.B., Quax P.H.A., Nossent A.Y. (2018). Adenosine-to-Inosine Editing of MicroRNA-487b Alters Target Gene Selection After Ischemia and Promotes Neovascularization. Circ. Res..

[28] Kume H., Hino K., Galipon J., Ui-Tei K. (2014). A-to-I editing in the miRNA seed region regulates target mRNA selection and silencing efficiency. Nucleic Acids Res..

[29] Liang H., Landweber L.F. (2007). Hypothesis: RNA editing of microRNA target sites in humans?. RNA.

[30] Nigita G., Veneziano D., Ferro A. (2015). A-to-I RNA Editing: Current Knowledge Sources and Computational Approaches with Special Emphasis on Non-Coding RNA Molecules. Front. Bioeng. Biotechnol..

[31] Ivanov A., Memczak S., Wyler E., Torti F., Porath H.T., Orejuela M.R., Piechotta M., Levanon E.Y., Landthaler M., Dieterich C., Rajewsky N. (2015). Analysis of intron sequences reveals hallmarks of circular RNA biogenesis in animals. Cell Rep..

[32] Breen M.S., Dobbyn A., Li Q., Roussos P., Hoffman G.E., Stahl E., Chess A., Sklar P., Li J.B., Devlin B. (2019). Global landscape and genetic regulation of RNA editing in cortical samples from individuals with schizophrenia. Nat. Neurosci..

[33] Park E., Jiang Y., Hao L., Hui J., Xing Y. (2021). Genetic variation and microRNA targeting of A-to-I RNA editing fine tune human tissue transcriptomes. Genome Biol..

[34] Quinones-Valdez G., Tran S.S., Jun H.I., Bahn J.H., Yang E.W., Zhan L., Brümmer A., Wei X., Van Nostrand E.L., Pratt G.A. (2019). Regulation of RNA editing by RNA-binding proteins in human cells. Commun. Biol..

[35] Cai D., Behrmann O., Hufert F., Dame G., Urban G. (2018). Direct DNA and RNA detection from large volumes of whole human blood. Sci. Rep..

[36] Kondratov K., Kurapeev D., Popov M., Sidorova M., Minasian S., Galagudza M., Kostareva A., Fedorov A. (2016). Heparinase treatment of heparin-contaminated plasma from coronary artery bypass grafting patients enables reliable quantification of microRNAs. Biomol. Detect. Quantif..

[37] Kirschner M.B., Edelman J.J.B., Kao S.C.H., Vallely M.P., van Zandwijk N., Reid G. (2013). The Impact of Hemolysis on Cell-Free microRNA Biomarkers. Front. Genet..

[38] Stojkovic S., Nossent A.Y., Haller P., Jäger B., Vargas K.G., Wojta J., Huber K. (2019). MicroRNAs as Regulators and Biomarkers of Platelet Function and Activity in Coronary Artery Disease. Thromb. Haemostasis.

[39] Evers D.L., Fowler C.B., Cunningham B.R., Mason J.T., O'Leary T.J. (2011). The effect of formaldehyde fixation on RNA: optimization of formaldehyde adduct removal. J. Mol. Diagn..

[40] Phan H.V., van Gent M., Drayman N., Basu A., Gack M.U., Tay S. (2021). High-throughput RNA sequencing of paraformaldehyde-fixed single cells. Nat. Commun..

[41] Vilades D., Martínez-Camblor P., Ferrero-Gregori A., Bär C., Lu D., Xiao K., Vea À., Nasarre L., Sanchez Vega J., Leta R. (2020). Plasma circular RNA hsa_circ_0001445 and coronary artery disease: Performance as a biomarker. Faseb. J..

[42] Lakkisto P., Dalgaard L.T., Belmonte T., Pinto-Sietsma S.J., Devaux Y., de Gonzalo-Calvo D., EU-CardioRNA COST Action CA17129 (2023). Development of circulating microRNA-based biomarkers for medical decision-making: a friendly reminder of what should NOT be done. Crit. Rev. Clin. Lab Sci..

[43] Görgens A., Corso G., Hagey D.W., Jawad Wiklander R., Gustafsson M.O., Felldin U., Lee Y., Bostancioglu R.B., Sork H., Liang X. (2022). Identification of storage conditions stabilizing extracellular vesicles preparations. J. Extracell. Vesicles.

[44] Chomczynski P., Sacchi N. (1987). Single-step method of RNA isolation by acid guanidinium thiocyanate-phenol-chloroform extraction. Anal. Biochem..

[45] Chomczynski P., Sacchi N. (2006). The single-step method of RNA isolation by acid guanidinium thiocyanate-phenol-chloroform extraction: twenty-something years on. Nat. Protoc..

[46] Mutiu A.I., Brandl C.J. (2005). RNA isolation from yeast using silica matrices. J. Biomol. Tech..

[47] Berensmeier S. (2006). Magnetic particles for the separation and purification of nucleic acids. Appl. Microbiol. Biotechnol..

[48] Rodriguez-Molina J.B., Turtola M. (2022). Birth of a poly(A) tail: mechanisms and control of mRNA polyadenylation. FEBS Open Bio.

[49] Zhang Y., Yang L., Chen L.L. (2014). Life without A tail: new formats of long noncoding RNAs. Int. J. Biochem. Cell Biol..

[50] Hrdlickova R., Toloue M., Tian B. (2017).

[51] Kraus A.J., Brink B.G., Siegel T.N. (2019). Efficient and specific oligo-based depletion of rRNA. Sci. Rep..

[52] Archer S.K., Shirokikh N.E., Preiss T. (2015). Probe-Directed Degradation (PDD) for Flexible Removal of Unwanted cDNA Sequences from RNA-Seq Libraries. Curr. Protoc. Hum. Genet..

[53] Arnaud O., Kato S., Poulain S., Plessy C. (2016). Targeted reduction of highly abundant transcripts using pseudo-random primers. Biotechniques.

[54] Nicholson A.W. (2014).

[55] Zhao S., Zhang Y., Gordon W., Quan J., Xi H., Du S., von Schack D., Zhang B. (2015). Comparison of stranded and non-stranded RNA-seq transcriptome profiling and investigation of gene overlap. BMC Genom..

[56] Liao J., Wang J., Liu Y., Li J., Duan L. (2019). Transcriptome sequencing of lncRNA, miRNA, mRNA and interaction network constructing in coronary heart disease. BMC Med. Genom..

[57] Yang I.S., Kim S. (2015). Analysis of Whole Transcriptome Sequencing Data: Workflow and Software. Genomics Inform..

[58] Xiao M.S., Wilusz J.E. (2019). An improved method for circular RNA purification using RNase R that efficiently removes linear RNAs containing G-quadruplexes or structured 3' ends. Nucleic Acids Res..

[59] Benesova S., Kubista M., Valihrach L. (2021). Small RNA-Sequencing: Approaches and Considerations for miRNA Analysis. Diagnostics.

[60] Fu Y., Wu P.H., Beane T., Zamore P.D., Weng Z. (2018). Elimination of PCR duplicates in RNA-seq and small RNA-seq using unique molecular identifiers. BMC Genom..

[61] Fuchs R.T., Sun Z., Zhuang F., Robb G.B. (2015). Bias in ligation-based small RNA sequencing library construction is determined by adaptor and RNA structure. PLoS One.

[62] Wright C., Rajpurohit A., Burke E.E., Williams C., Collado-Torres L., Kimos M., Brandon N.J., Cross A.J., Jaffe A.E., Weinberger D.R., Shin J.H. (2019). Comprehensive assessment of multiple biases in small RNA sequencing reveals significant differences in the performance of widely used methods. BMC Genom..

[63] Mercer T.R., Clark M.B., Crawford J., Brunck M.E., Gerhardt D.J., Taft R.J., Nielsen L.K., Dinger M.E., Mattick J.S. (2014). Targeted sequencing for gene discovery and quantification using RNA CaptureSeq. Nat. Protoc..

[64] Clark M.B., Mercer T.R., Bussotti G., Leonardi T., Haynes K.R., Crawford J., Brunck M.E., Cao K.A.L., Thomas G.P., Chen W.Y. (2015). Quantitative gene profiling of long noncoding RNAs with targeted RNA sequencing. Nat. Methods.

[65] Portal M.M., Pavet V., Erb C., Gronemeyer H. (2015). TARDIS, a targeted RNA directional sequencing method for rare RNA discovery. Nat. Protoc..

[66] Halvardson J., Zaghlool A., Feuk L. (2013). Exome RNA sequencing reveals rare and novel alternative transcripts. Nucleic Acids Res..

[67] Cieslik M., Chugh R., Wu Y.M., Wu M., Brennan C., Lonigro R., Su F., Wang R., Siddiqui J., Mehra R. (2015). The use of exome capture RNA-seq for highly degraded RNA with application to clinical cancer sequencing. Genome Res..

[68] Samorodnitsky E., Jewell B.M., Hagopian R., Miya J., Wing M.R., Lyon E., Damodaran S., Bhatt D., Reeser J.W., Datta J., Roychowdhury S. (2015). Evaluation of Hybridization Capture Versus Amplicon-Based Methods for Whole-Exome Sequencing. Hum. Mutat..

[69] Zhang R., Li X., Ramaswami G., Smith K.S., Turecki G., Montgomery S.B., Li J.B. (2014). Quantifying RNA allelic ratios by microfluidic multiplex PCR and sequencing. Nat. Methods.

[70] Zaidan H., Ramaswami G., Barak M., Li J.B., Gaisler-Salomon I. (2018). Pre-reproductive stress and fluoxetine treatment in rats affect offspring A-to-I RNA editing, gene expression and social behavior. Environ. Epigenet..

[71] Khozyainova A.A., Valyaeva A.A., Arbatsky M.S., Isaev S.V., Iamshchikov P.S., Volchkov E.V., Sabirov M.S., Zainullina V.R., Chechekhin V.I., Vorobev R.S. (2023). Complex Analysis of Single-Cell RNA Sequencing Data. Biochemistry.

[72] Wehrens M., de Leeuw A.E., Wright-Clark M., Eding J.E.C., Boogerd C.J., Molenaar B., van der Kraak P.H., Kuster D.W.D., van der Velden J., Michels M. (2022). Single-cell transcriptomics provides insights into hypertrophic cardiomyopathy. Cell Rep..

[73] Bonacina F., Di Costanzo A., Genkel V., Kong X.Y., Kroon J., Stimjanin E., Tsiantoulas D., Grootaert M.O. (2023). The heterogeneous cellular landscape of atherosclerosis: Implications for future research and therapies. A collaborative review from the EAS young fellows. Atherosclerosis.

[74] Pettas S., Karagianni K., Kanata E., Chatziefstathiou A., Christoudia N., Xanthopoulos K., Sklaviadis T., Dafou D. (2022). Profiling Microglia through Single-Cell RNA Sequencing over the Course of Development, Aging, and Disease. Cells.

[75] Zhao L., Huang W., Yi S. (2023). Cellular complexity of the peripheral nervous system: Insights from single-cell resolution. Front. Neurosci..

[76] Gal-Mark N., Shallev L., Sweetat S., Barak M., Billy Li J., Levanon E.Y., Eisenberg E., Behar O. (2017). Abnormalities in A-to-I RNA editing patterns in CNS injuries correlate with dynamic changes in cell type composition. Sci. Rep..

[77] Lundin E., Wu C., Widmark A., Behm M., Hjerling-Leffler J., Daniel C., Öhman M., Nilsson M. (2020). Spatiotemporal mapping of RNA editing in the developing mouse brain using in situ sequencing reveals regional and cell-type-specific regulation. BMC Biol..

[78] Sapiro A.L., Shmueli A., Henry G.L., Li Q., Shalit T., Yaron O., Paas Y., Billy Li J., Shohat-Ophir G. (2019). Illuminating spatial A-to-I RNA editing signatures within the Drosophila brain. Proc. Natl. Acad. Sci. USA.

[79] Cuddleston W.H., Li J., Fan X., Kozenkov A., Lalli M., Khalique S., Dracheva S., Mukamel E.A., Breen M.S. (2022). Cellular and genetic drivers of RNA editing variation in the human brain. Nat. Commun..

[80] Islam S., Zeisel A., Joost S., La Manno G., Zajac P., Kasper M., Lönnerberg P., Linnarsson S. (2014). Quantitative single-cell RNA-seq with unique molecular identifiers. Nat. Methods.

[81] Deng Q., Ramsköld D., Reinius B., Sandberg R. (2014). Single-cell RNA-seq reveals dynamic, random monoallelic gene expression in mammalian cells. Science.

[82] Kowalczyk M.S., Tirosh I., Heckl D., Rao T.N., Dixit A., Haas B.J., Schneider R.K., Wagers A.J., Ebert B.L., Regev A. (2015). Single-cell RNA-seq reveals changes in cell cycle and differentiation programs upon aging of hematopoietic stem cells. Genome Res..

[83] Wu Y., Hao S., Xu X., Dong G., Ouyang W., Liu C., Sun H.X. (2023). A novel computational method enables RNA editome profiling during human hematopoiesis from scRNA-seq data. Sci. Rep..

[84] Adewale B.A. (2020). Will long-read sequencing technologies replace short-read sequencing technologies in the next 10 years?. Afr. J. Lab. Med..

[85] Lucas M.C., Novoa E.M. (2023). Long-read sequencing in the era of epigenomics and epitranscriptomics. Nat. Methods.

[86] Eid J., Fehr A., Gray J., Luong K., Lyle J., Otto G., Peluso P., Rank D., Baybayan P., Bettman B. (2009). Real-time DNA sequencing from single polymerase molecules. Science.

[87] Garalde D.R., Snell E.A., Jachimowicz D., Sipos B., Lloyd J.H., Bruce M., Pantic N., Admassu T., James P., Warland A. (2018). Highly parallel direct RNA sequencing on an array of nanopores. Nat. Methods.

[88] Furlan M., Delgado-Tejedor A., Mulroney L., Pelizzola M., Novoa E.M., Leonardi T. (2021). Computational methods for RNA modification detection from nanopore direct RNA sequencing data. RNA Biol..

[89] Nguyen T.A., Heng J.W.J., Kaewsapsak P., Kok E.P.L., Stanojević D., Liu H., Cardilla A., Praditya A., Yi Z., Lin M. (2022). Direct identification of A-to-I editing sites with nanopore native RNA sequencing. Nat. Methods.

[90] (2022). Deep learning identifies A-to-I RNA edits using nanopore sequencing data. Nat. Methods.

[91] Chen L., Ou L., Jing X., Kong Y., Xie B., Zhang N., Shi H., Qin H., Li X., Hao P. (2023). DeepEdit: single-molecule detection and phasing of A-to-I RNA editing events using nanopore direct RNA sequencing. Genome Biol..

[92] Diroma M.A., Ciaccia L., Pesole G., Picardi E. (2019). Elucidating the editome: bioinformatics approaches for RNA editing detection. Brief. Bioinform..

[93] Lee J.H., Ang J.K., Xiao X. (2013). Analysis and design of RNA sequencing experiments for identifying RNA editing and other single-nucleotide variants. RNA.

[94] Bahn J.H., Lee J.H., Li G., Greer C., Peng G., Xiao X. (2012). Accurate identification of A-to-I RNA editing in human by transcriptome sequencing. Genome Res..

[95] Light D., Haas R., Yazbak M., Elfand T., Blau T., Lamm A.T. (2021). RESIC: A Tool for Comprehensive Adenosine to Inosine RNA Editing Site Identification and Classification. Front. Genet..

[96] Picardi E., Pesole G. (2013). REDItools: high-throughput RNA editing detection made easy. Bioinformatics.

[97] John D., Weirick T., Dimmeler S., Uchida S. (2017). RNAEditor: easy detection of RNA editing events and the introduction of editing islands. Brief. Bioinform..

[98] Li H., Durbin R. (2009). Fast and accurate short read alignment with Burrows-Wheeler transform. Bioinformatics.

[99] Sander J., Ester M., Kriegel H.-P., Xu X. (1998). Density-Based Clustering in Spatial Databases: The Algorithm GDBSCAN and Its Applications. Data Min. Knowl. Discov..

[100] Wang Z., Lian J., Li Q., Zhang P., Zhou Y., Zhan X., Zhang G. (2016). RES-Scanner: a software package for genome-wide identification of RNA-editing sites. GigaScience.

[101] Alon S., Eisenberg E. (2013). Identifying RNA editing sites in miRNAs by deep sequencing. Methods Mol. Biol..

[102] Xiong H., Liu D., Li Q., Lei M., Xu L., Wu L., Wang Z., Ren S., Li W., Xia M. (2017). RED-ML: a novel, effective RNA editing detection method based on machine learning. GigaScience.

[103] Zhang Q., Xiao X. (2015). Genome sequence-independent identification of RNA editing sites. Nat. Methods.

[104] Liu Z., Quinones-Valdez G., Fu T., Huang E., Choudhury M., Reese F., Mortazavi A., Xiao X. (2023). L-GIREMI uncovers RNA editing sites in long-read RNA-seq. Genome Biol..

[105] Piechotta M., Wyler E., Ohler U., Landthaler M., Dieterich C. (2017). JACUSA: site-specific identification of RNA editing events from replicate sequencing data. BMC Bioinf..

[106] Piechotta M., Naarmann-de Vries I.S., Wang Q., Altmüller J., Dieterich C. (2022). RNA modification mapping with JACUSA2. Genome Biol..

[107] Zhang F., Lu Y., Yan S., Xing Q., Tian W. (2017). SPRINT: an SNP-free toolkit for identifying RNA editing sites. Bioinformatics.

[108] Kim D.D.Y., Kim T.T.Y., Walsh T., Kobayashi Y., Matise T.C., Buyske S., Gabriel A. (2004). Widespread RNA editing of embedded alu elements in the human transcriptome. Genome Res..

[109] Barak M., Levanon E.Y., Eisenberg E., Paz N., Rechavi G., Church G.M., Mehr R. (2009). Evidence for large diversity in the human transcriptome created by Alu RNA editing. Nucleic Acids Res..

[110] Blow M., Futreal P.A., Wooster R., Stratton M.R. (2004). A survey of RNA editing in human brain. Genome Res..

[111] Carmi S., Borukhov I., Levanon E.Y. (2011). Identification of widespread ultra-edited human RNAs. PLoS Genet..

[112] Quiles-Jiménez A., Gregersen I., Mittelstedt Leal de Sousa M., Abbas A., Kong X.Y., Alseth I., Holm S., Dahl T.B., Skagen K., Skjelland M. (2020). N6-methyladenosine in RNA of atherosclerotic plaques: An epitranscriptomic signature of human carotid atherosclerosis. Biochem. Biophys. Res. Commun..

[113] Alon S., Erew M., Eisenberg E. (2015). DREAM: a webserver for the identification of editing sites in mature miRNAs using deep sequencing data. Bioinformatics.

[114] Yao L., Wang H., Song Y., Dai Z., Yu H., Yin M., Wang D., Yang X., Wang J., Wang T. (2019). Large-scale prediction of ADAR-mediated effective human A-to-I RNA editing. Brief. Bioinform..

[115] Nigita G., Alaimo S., Ferro A., Giugno R., Pulvirenti A. (2015). Knowledge in the Investigation of A-to-I RNA Editing Signals. Front. Bioeng. Biotechnol..

[116] Niu G., Zou D., Li M., Zhang Y., Sang J., Xia L., Li M., Liu L., Cao J., Zhang Y. (2019). Editome Disease Knowledgebase (EDK): a curated knowledgebase of editome-disease associations in human. Nucleic Acids Res..

[117] Zhu H., Huang L., Liu S., Dai Z., Songyang Z., Weng Z., Xiong Y. (2022). REIA: A database for cancer A-to-I RNA editing with interactive analysis. Int. J. Biol. Sci..

[118] Picardi E., D'Erchia A.M., Lo Giudice C., Pesole G. (2017). REDIportal: a comprehensive database of A-to-I RNA editing events in humans. Nucleic Acids Res..

[119] Lin C.H., Chen S.C.C. (2019). The Cancer Editome Atlas: A Resource for Exploratory Analysis of the Adenosine-to-Inosine RNA Editome in Cancer. Cancer Res..

[120] Stephens Z.D., Lee S.Y., Faghri F., Campbell R.H., Zhai C., Efron M.J., Iyer R., Schatz M.C., Sinha S., Robinson G.E. (2015). Big Data: Astronomical or Genomical?. PLoS Biol..

[121] Gong J., Liu C., Liu W., Xiang Y., Diao L., Guo A.Y., Han L. (2017). LNCediting: a database for functional effects of RNA editing in lncRNAs. Nucleic Acids Res..

[122] Liu X., Jian X., Boerwinkle E. (2011). dbNSFP: a lightweight database of human nonsynonymous SNPs and their functional predictions. Hum. Mutat..

[123] Kennedy B., Kronenberg Z., Hu H., Moore B., Flygare S., Reese M.G., Jorde L.B., Yandell M., Huff C. (2014). Using VAAST to Identify Disease-Associated Variants in Next-Generation Sequencing Data. Curr. Protoc. Hum. Genet..

[124] Coonrod E.M., Margraf R.L., Russell A., Voelkerding K.V., Reese M.G. (2013). Clinical analysis of genome next-generation sequencing data using the Omicia platform. Expert Rev. Mol. Diagn..

[125] Vandeweyer G., Van Laer L., Loeys B., Van den Bulcke T., Kooy R.F. (2014). VariantDB: a flexible annotation and filtering portal for next generation sequencing data. Genome Med..

[126] Yandell M., Huff C., Hu H., Singleton M., Moore B., Xing J., Jorde L.B., Reese M.G. (2011). A probabilistic disease-gene finder for personal genomes. Genome Res..

[127] Wang K., Li M., Hakonarson H. (2010). ANNOVAR: functional annotation of genetic variants from high-throughput sequencing data. Nucleic Acids Res..

[128] Cingolani P., Platts A., Wang L.L., Coon M., Nguyen T., Wang L., Land S.J., Lu X., Ruden D.M. (2012). A program for annotating and predicting the effects of single nucleotide polymorphisms, SnpEff: SNPs in the genome of Drosophila melanogaster strain w1118; iso-2; iso-3. Fly (Austin).

[129] Pagel K.A., Kim R., Moad K., Busby B., Zheng L., Tokheim C., Ryan M., Karchin R. (2020). Integrated Informatics Analysis of Cancer-Related Variants. JCO Clin. Cancer Inform..

[130] McLaren W., Gil L., Hunt S.E., Riat H.S., Ritchie G.R.S., Thormann A., Flicek P., Cunningham F. (2016). The Ensembl Variant Effect Predictor. Genome Biol..

[131] Shannon P., Markiel A., Ozier O., Baliga N.S., Wang J.T., Ramage D., Amin N., Schwikowski B., Ideker T. (2003). Cytoscape: a software environment for integrated models of biomolecular interaction networks. Genome Res..

[132] Reimand J., Kull M., Peterson H., Hansen J., Vilo J. (2007). g:Profiler--a web-based toolset for functional profiling of gene lists from large-scale experiments. Nucleic Acids Res..

[133] Kuleshov M.V., Jones M.R., Rouillard A.D., Fernandez N.F., Duan Q., Wang Z., Koplev S., Jenkins S.L., Jagodnik K.M., Lachmann A. (2016). Enrichr: a comprehensive gene set enrichment analysis web server 2016 update. Nucleic Acids Res..

[134] Huang D.W., Sherman B.T., Lempicki R.A. (2009). Systematic and integrative analysis of large gene lists using DAVID bioinformatics resources. Nat. Protoc..

[135] Chen J., Bardes E.E., Aronow B.J., Jegga A.G. (2009). ToppGene Suite for gene list enrichment analysis and candidate gene prioritization. Nucleic Acids Res..

[136] Mi H., Muruganujan A., Thomas P.D. (2013). PANTHER in 2013: modeling the evolution of gene function, and other gene attributes, in the context of phylogenetic trees. Nucleic Acids Res..

[137] Subramanian A., Tamayo P., Mootha V.K., Mukherjee S., Ebert B.L., Gillette M.A., Paulovich A., Pomeroy S.L., Golub T.R., Lander E.S., Mesirov J.P. (2005). Gene set enrichment analysis: a knowledge-based approach for interpreting genome-wide expression profiles. Proc. Natl. Acad. Sci. USA.

[138] Merico D., Isserlin R., Stueker O., Emili A., Bader G.D. (2010). Enrichment map: a network-based method for gene-set enrichment visualization and interpretation. PLoS One.

[139] Zhou Y., Zhou B., Pache L., Chang M., Khodabakhshi A.H., Tanaseichuk O., Benner C., Chanda S.K. (2019). Metascape provides a biologist-oriented resource for the analysis of systems-level datasets. Nat. Commun..

[140] Xie Z., Bailey A., Kuleshov M.V., Clarke D.J.B., Evangelista J.E., Jenkins S.L., Lachmann A., Wojciechowicz M.L., Kropiwnicki E., Jagodnik K.M. (2021). Gene Set Knowledge Discovery with Enrichr. Curr. Protoc..

[141] Garcia-Moreno A., Carmona-Saez P. (2020). Computational Methods and Software Tools for Functional Analysis of miRNA Data. Biomolecules.

[142] Kozomara A., Birgaoanu M., Griffiths-Jones S. (2019). miRBase: from microRNA sequences to function. Nucleic Acids Res..

[143] Vlachos I.S., Paraskevopoulou M.D., Karagkouni D., Georgakilas G., Vergoulis T., Kanellos I., Anastasopoulos I.L., Maniou S., Karathanou K., Kalfakakou D. (2015). DIANA-TarBase v7.0: indexing more than half a million experimentally supported miRNA:mRNA interactions. Nucleic Acids Res..

[144] Georgakilas G., Vlachos I.S., Zagganas K., Vergoulis T., Paraskevopoulou M.D., Kanellos I., Tsanakas P., Dellis D., Fevgas A., Dalamagas T., Hatzigeorgiou A.G. (2016). DIANA-miRGen v3.0: accurate characterization of microRNA promoters and their regulators. Nucleic Acids Res..

[145] Backes C., Fehlmann T., Kern F., Kehl T., Lenhof H.P., Meese E., Keller A. (2018). miRCarta: a central repository for collecting miRNA candidates. Nucleic Acids Res..

[146] Fromm B., Domanska D., Høye E., Ovchinnikov V., Kang W., Aparicio-Puerta E., Johansen M., Flatmark K., Mathelier A., Hovig E. (2020). MirGeneDB 2.0: the metazoan microRNA complement. Nucleic Acids Res..

[147] Paraskevopoulou M.D., Georgakilas G., Kostoulas N., Vlachos I.S., Vergoulis T., Reczko M., Filippidis C., Dalamagas T., Hatzigeorgiou A.G. (2013). DIANA-microT web server v5.0: service integration into miRNA functional analysis workflows. Nucleic Acids Res..

[148] Sticht C., De La Torre C., Parveen A., Gretz N. (2018). miRWalk: An online resource for prediction of microRNA binding sites. PLoS One.

[149] Vlachos I.S., Zagganas K., Paraskevopoulou M.D., Georgakilas G., Karagkouni D., Vergoulis T., Dalamagas T., Hatzigeorgiou A.G. (2015). DIANA-miRPath v3.0: deciphering microRNA function with experimental support. Nucleic Acids Res..

[150] Vlachos I.S., Vergoulis T., Paraskevopoulou M.D., Lykokanellos F., Georgakilas G., Georgiou P., Chatzopoulos S., Karagkouni D., Christodoulou F., Dalamagas T., Hatzigeorgiou A.G. (2016). DIANA-mirExTra v2.0: Uncovering microRNAs and transcription factors with crucial roles in NGS expression data. Nucleic Acids Res..

[151] Agarwal V., Bell G.W., Nam J.W., Bartel D.P. (2015). Predicting effective microRNA target sites in mammalian mRNAs. Elife.

[152] Ding J., Li X., Hu H. (2016). TarPmiR: a new approach for microRNA target site prediction. Bioinformatics.

[153] Volders P.J., Verheggen K., Menschaert G., Vandepoele K., Martens L., Vandesompele J., Mestdagh P. (2015). An update on LNCipedia: a database for annotated human lncRNA sequences. Nucleic Acids Res..

[154] Bhartiya D., Pal K., Ghosh S., Kapoor S., Jalali S., Panwar B., Jain S., Sati S., Sengupta S., Sachidanandan C. (2013). lncRNome: A Comprehensive Knowledgebase of Human Long Noncoding RNAs. Database.

[155] Paraskevopoulou M.D., Vlachos I.S., Karagkouni D., Georgakilas G., Kanellos I., Vergoulis T., Zagganas K., Tsanakas P., Floros E., Dalamagas T., Hatzigeorgiou A.G. (2016). DIANA-LncBase v2: indexing microRNA targets on non-coding transcripts. Nucleic Acids Res..

[156] Lerner T., Kluesner M., Tasakis R.N., Moriarity B.S., Papavasiliou F.N., Pecori R. (2021). C-to-U RNA Editing: From Computational Detection to Experimental Validation. Methods Mol. Biol..

[157] Androvic P., Valihrach L., Elling J., Sjoback R., Kubista M. (2017). Two-tailed RT-qPCR: a novel method for highly accurate miRNA quantification. Nucleic Acids Res..

[158] Voss G., Ceder Y. (2023). Two-Tailed RT-qPCR for the Quantification of A-to-I-Edited microRNA Isoforms. Curr. Protoc..

[159] Bhakta S., Sakari M., Tsukahara T. (2020). RNA editing of BFP, a point mutant of GFP, using artificial APOBEC1 deaminase to restore the genetic code. Sci. Rep..

[160] Dick A.L.W., Khermesh K., Paul E., Stamp F., Levanon E.Y., Chen A. (2019). Adenosine-to-Inosine RNA Editing Within Corticolimbic Brain Regions Is Regulated in Response to Chronic Social Defeat Stress in Mice. Front. Psychiatry.

[161] Paul D., Sinha A.N., Ray A., Lal M., Nayak S., Sharma A., Mehani B., Mukherjee D., Laddha S.V., Suri A. (2017). A-to-I editing in human miRNAs is enriched in seed sequence, influenced by sequence contexts and significantly hypoedited in glioblastoma multiforme. Sci. Rep..

[162] Jain M., Weber A., Maly K., Manjaly G., Deek J., Tsvyetkova O., Stulić M., Toca-Herrera J.L., Jantsch M.F. (2022). A-to-I RNA editing of Filamin A regulates cellular adhesion, migration and mechanical properties. FEBS J..

[163] Kokot K.E., Kneuer J.M., John D., Rebs S., Möbius-Winkler M.N., Erbe S., Müller M., Andritschke M., Gaul S., Sheikh B.N. (2022). Reduction of A-to-I RNA editing in the failing human heart regulates formation of circular RNAs. Basic Res. Cardiol..

[164] Tian N., Li X., Luo Y., Han Z., Li Z., Fan C. (2014). Curcumin regulates the metabolism of low density lipoproteins by improving the C-to-U RNA editing efficiency of apolipoprotein B in primary rat hepatocytes. Mol. Med. Rep..

[165] Mukherjee P., Raghava Kurup R., Hundley H.A. (2021). RNA immunoprecipitation to identify in vivo targets of RNA editing and modifying enzymes. Methods Enzymol..

[166] Thomas J.M., Beal P.A. (2017). How do ADARs bind RNA? New protein-RNA structures illuminate substrate recognition by the RNA editing ADARs. Bioessays.

[167] Dafou D., Kanata E., Pettas S., Bekas N., Dimitriadis A., Kempapidou G., Lagoudaki R., Theotokis P., Touloumi O., Delivanoglou N. (2022).

[168] Merkle T., Merz S., Reautschnig P., Blaha A., Li Q., Vogel P., Wettengel J., Li J.B., Stafforst T. (2019). Precise RNA editing by recruiting endogenous ADARs with antisense oligonucleotides. Nat. Biotechnol..

[169] Katrekar D., Chen G., Meluzzi D., Ganesh A., Worlikar A., Shih Y.R., Varghese S., Mali P. (2019). In vivo RNA editing of point mutations via RNA-guided adenosine deaminases. Nat. Methods.

[170] Yi Z., Qu L., Tang H., Liu Z., Liu Y., Tian F., Wang C., Zhang X., Feng Z., Yu Y. (2022). Engineered circular ADAR-recruiting RNAs increase the efficiency and fidelity of RNA editing in vitro and in vivo. Nat. Biotechnol..

[171] Jain M., Manjaly G., Maly K., de Vries M.R., Janisiw M., König L., Nossent A.Y., Jantsch M.F. (2022). Filamin A pre-mRNA editing modulates vascularization and tumor growth. Mol. Ther. Nucleic Acids.

[172] Khermesh K., D'Erchia A.M., Barak M., Annese A., Wachtel C., Levanon E.Y., Picardi E., Eisenberg E. (2016). Reduced levels of protein recoding by A-to-I RNA editing in Alzheimer's disease. RNA.

[173] Vlachogiannis N.I., Sachse M., Georgiopoulos G., Zormpas E., Bampatsias D., Delialis D., Bonini F., Galyfos G., Sigala F., Stamatelopoulos K. (2021). Adenosine-to-inosine Alu RNA editing controls the stability of the pro-inflammatory long noncoding RNA NEAT1 in atherosclerotic cardiovascular disease. J. Mol. Cell. Cardiol..

[174] Altaf F., Vesely C., Sheikh A.M., Munir R., Shah S.T.A., Tariq A. (2019). Modulation of ADAR mRNA expression in patients with congenital heart defects. PLoS One.

[175] Ma Y., Dammer E.B., Felsky D., Duong D.M., Klein H.U., White C.C., Zhou M., Logsdon B.A., McCabe C., Xu J. (2021). Atlas of RNA editing events affecting protein expression in aged and Alzheimer's disease human brain tissue. Nat. Commun..

[176] Kanata E., Llorens F., Dafou D., Dimitriadis A., Thüne K., Xanthopoulos K., Bekas N., Espinosa J.C., Schmitz M., Marín-Moreno A. (2019). RNA editing alterations define manifestation of prion diseases. Proc. Natl. Acad. Sci. USA.

[177] Hosaka T., Tsuji H., Kwak S. (2021). RNA Editing: A New Therapeutic Target in Amyotrophic Lateral Sclerosis and Other Neurological Diseases. Int. J. Mol. Sci..

[178] Decher N., Streit A.K., Rapedius M., Netter M.F., Marzian S., Ehling P., Schlichthörl G., Craan T., Renigunta V., Köhler A. (2010). RNA editing modulates the binding of drugs and highly unsaturated fatty acids to the open pore of Kv potassium channels. EMBO J..

[179] Mandrekar J.N. (2010). Receiver operating characteristic curve in diagnostic test assessment. J. Thorac. Oncol..

[180] Salvetat N., Checa-Robles F.J., Patel V., Cayzac C., Dubuc B., Chimienti F., Abraham J.D., Dupré P., Vetter D., Méreuze S. (2022). A game changer for bipolar disorder diagnosis using RNA editing-based biomarkers. Transl. Psychiatry.

[181] Sinnamon J.R., Kim S.Y., Corson G.M., Song Z., Nakai H., Adelman J.P., Mandel G. (2017). Site-directed RNA repair of endogenous Mecp2 RNA in neurons. Proc. Natl. Acad. Sci. USA.

[182] Wu X., Wang L., Wang K., Li J., Chen R., Wu X., Ni G., Liu C., Das S., Sluijter J.P.G. (2022). ADAR2 increases in exercised heart and protects against myocardial infarction and doxorubicin-induced cardiotoxicity. Mol. Ther..

[183] Goossens E.A.C., de Vries M.R., Simons K.H., Putter H., Quax P.H.A., Nossent A.Y. (2019). miRMap: Profiling 14q32 microRNA Expression and DNA Methylation Throughout the Human Vasculature. Front. Cardiovasc. Med..

[184] Rogg E.M., Abplanalp W.T., Bischof C., John D., Schulz M.H., Krishnan J., Fischer A., Poluzzi C., Schaefer L., Bonauer A. (2018). Analysis of Cell Type-Specific Effects of MicroRNA-92a Provides Novel Insights Into Target Regulation and Mechanism of Action. Circulation.

